# Electronic structure of strongly correlated systems: recent developments in multiconfiguration pair-density functional theory and multiconfiguration nonclassical-energy functional theory

**DOI:** 10.1039/d2sc01022d

**Published:** 2022-06-07

**Authors:** Chen Zhou, Matthew R. Hermes, Dihua Wu, Jie J. Bao, Riddhish Pandharkar, Daniel S. King, Dayou Zhang, Thais R. Scott, Aleksandr O. Lykhin, Laura Gagliardi, Donald G. Truhlar

**Affiliations:** Department of Chemistry, Chemical Theory Center, Minnesota Supercomputing Institute, University of Minnesota 207 Pleasant Street SE Minneapolis MN 55455-0431 USA truhlar@umn.edu; Department of Chemistry, Pritzker School of Molecular Engineering, The James Franck Institute and Chicago Center for Theoretical Chemistry, The University of Chicago Chicago IL 60637 USA lgagliardi@uchicago.edu; Argonne National Laboratory Lemont Illinois 60439 USA

## Abstract

Strong electron correlation plays an important role in transition-metal and heavy-metal chemistry, magnetic molecules, bond breaking, biradicals, excited states, and many functional materials, but it provides a significant challenge for modern electronic structure theory. The treatment of strongly correlated systems usually requires a multireference method to adequately describe spin densities and near-degeneracy correlation. However, quantitative computation of dynamic correlation with multireference wave functions is often difficult or impractical. Multiconfiguration pair-density functional theory (MC-PDFT) provides a way to blend multiconfiguration wave function theory and density functional theory to quantitatively treat both near-degeneracy correlation and dynamic correlation in strongly correlated systems; it is more affordable than multireference perturbation theory, multireference configuration interaction, or multireference coupled cluster theory and more accurate for many properties than Kohn–Sham density functional theory. This perspective article provides a brief introduction to strongly correlated systems and previously reviewed progress on MC-PDFT followed by a discussion of several recent developments and applications of MC-PDFT and related methods, including localized-active-space MC-PDFT, generalized active-space MC-PDFT, density-matrix-renormalization-group MC-PDFT, hybrid MC-PDFT, multistate MC-PDFT, spin–orbit coupling, analytic gradients, and dipole moments. We also review the more recently introduced multiconfiguration nonclassical-energy functional theory (MC-NEFT), which is like MC-PDFT but allows for other ingredients in the nonclassical-energy functional. We discuss two new kinds of MC-NEFT methods, namely multiconfiguration density coherence functional theory and machine-learned functionals.

## Abstract 2

This feature article overviews recent work on active spaces, matrix product reference states, treatment of quasidegeneracy, hybrid theory, density-coherence functionals, machine-learned functionals, spin–orbit coupling, gradients, and dipole moments.

## Graphical abstract

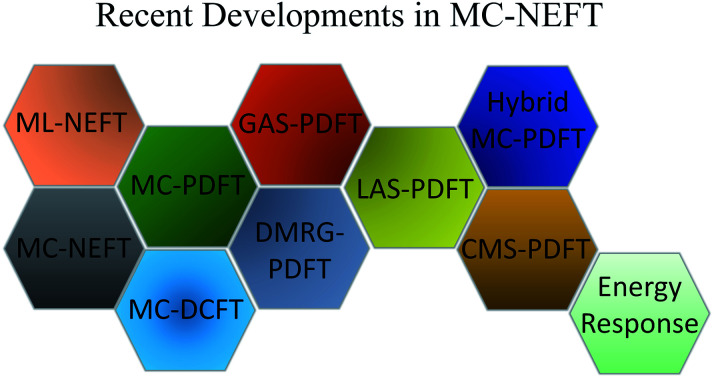

## 1. Introduction

Kohn–Sham density functional theory^1–4^ (KS theory) revolutionized the ability of computational chemists to apply high-quality quantum mechanical electronic structure theory to complex molecules and materials. The main advantage of KS theory over other approaches is the very high accuracy-to-cost ratio for many-body electronic structure problems. KS theory includes an existence theorem for a universal exchange–correlation functional that would make the ground-state energy and electron density exact for any chemical system. Still, there is no systematic way to obtain this functional, and it will probably never be found. There are, however, many approximate exchange–correlation functionals; they are typically functionals of up-spin and down-spin effective electron densities and other quantities like the electron density gradients or kinetic energy densities. With existing approximate functionals, KS theory is more accurate for weakly correlated systems than strongly correlated ones.^4–6^ To remedy this, we have proposed a new kind of density functional theory, called multiconfiguration nonclassical-energy functional theory (MC-NEFT), that is designed to treat strongly correlated systems more accurately. The most studied example of MC-NEFT is multiconfiguration pair-density functional theory^7^ (MC-PDFT). Because we have already reviewed the original work on pair-density functional theory^8,9^ and the whole group of theories that blend wave function theory with density functional theory for excited states,^10^ this perspective primarily focuses on more recent developments in MC-PDFT and provides a first review of other kinds of MC-NEFT. First, however, we introduce strongly correlated systems, MC-PDFT, and MC-NEFT.

The paper is organized into themes and sections as summarized in Table 1.

**Table 1:** Themes and sections

Section	Title
**Introductory remarks**	
1	Introduction
2	Strongly correlated systems
3	MC-PDFT and MC-NEFT

**Energies for large molecules and very large active spaces**	
4	LAS-PDFT, GAS-PDFT, and SP-PDFT
5	DMRG-PDFT

**Functional development**	
6	On-top functionals
7	Hybrid MC-PDFT
8	Multiconfiguration density coherence functional theory
9	Machine-learned functionals

**Going beyond the electronic ground state**	
10	Multistate pair-density functional theory
11	Spin–orbit coupling

**Analytic gradients and response properties**	
12	Forces by analytic gradients
13	Dipole moments

**Conclusion**	
14	Concluding remarks

## 2. Strongly correlated systems

The original use of the term “strongly correlated system” is tied to the idea of a reference function to be used as a zero-order wave function for many-body perturbation theory. The zero-order wave function in many-body perturbation theory is an antisymmetrized independent-particle wave function. An unsymmetrized independent-particle wave function would treat the electronic motions as uncorrelated; the two-particle probability density *ρ*(**r**_1_,*σ*_1_,**r**_2_,*σ*_2_) for finding one particle at position **r**_1_ with spin *σ*_1_ and another simultaneously at position **r**_2_ with spin *σ*_2_ would be just the product of one-particle probabilities *ρ*(**r**_1_,*σ*_1_) and *ρ*(**r**_2_,*σ*_2_). This issue is addressed by using an antisymmetrized zero-order wave function, for example, a Slater determinant composed of one-particle wave functions known as spin–orbitals; antisymmetrization introduces only the minimal amount of correlation necessary to satisfy the Pauli exclusion principle. The perturbation in many-body perturbation theory is the electron–electron interaction, which introduces the rest of the electron correlation. If the low orders of perturbation theory yield good accuracy, the system is said to be weakly correlated; if low orders of perturbation theory do not yield good accuracy, the system is said to be strongly correlated. Since “good accuracy” is vague, the border between strong and weak correlation is not precisely defined, but nevertheless, many systems are clearly on the strongly correlated side.

Reference functions are used not only in many-body perturbation theory but also in configuration interaction (CI) theory and coupled-cluster (CC) theory. For example, CI theory writes the wave function as a superposition of configuration state functions (CSFs, which may be either Slater determinants or linear combinations of them), where a configuration is a way of assigning electrons to spin–orbitals, and a CSF is a symmetry-adapted linear combination of Slater determinants with the same configuration for which the total electron spin *S* is a good quantum number, or it is a single Slater determinant. In CI theory, one generates CSFs by exciting electrons from occupied spin–orbitals in a reference function to those that are not occupied. Coupled-cluster theory is also based on excitations from a reference function. In strongly correlated systems, low-order excitations from only one reference CSF will not produce all necessary excitations with accurate enough coefficients to describe the true electronic wave function qualitatively. This issue is particularly prevalent when two or more CSFs are nearly degenerate (which in turn may result from orbital degeneracies). For this reason, strong correlation is often called near-degeneracy correlation. As already mentioned, the boundary between strongly correlated and weakly correlated is imprecise; instead of using perturbation theory, one may use the definition that a weakly correlated system is one for which chemical accuracy (usually defined as 1 kcal mol^−1^) can be obtained by coupled-cluster theory with single and double excitations and a perturbative treatment of connected triple excitations, and a strongly correlated system is one that requires higher excitations for chemical accuracy. The common way to overcome this issue without enumerating a combinatorially increasing number of high-order excitations from a single CSF is to generate low-order excitations from multiple CSFs (or a linear combination of CSFs), *i.e.*, multiple reference functions. For this reason, a molecule with strong correlation is often called a multireference system.

Strong correlation is also called static correlation, and correlation not due to near-degeneracy effects is sometimes called dynamic correlation. The above discussion gives another perspective that helps us identify whether a system is strongly correlated, *i.e.*, we ask whether there are near-degeneracy effects, *i.e.*, nearly degenerate CSFs, which are often due to nearly degenerate orbitals in partially filled subshells. We expect degeneracies and near-degeneracies in, for example, open-shell transition-metal compounds, molecular magnets, and most intermediates in transition-metal catalysis. The near degeneracy in such systems is due to half-filled valence d or f orbitals of weakly interacting metal centers. Biradicals constitute another class of multireference systems, and since dissociation of single bonds leads to biradicals, we expect significantly stretched single bonds to be strongly correlated. Finally, we note that although the ground electronic state of closed-shell molecules is usually widely separated from the excited states, the excited states themselves tend to form a dense manifold of states. Hence many (maybe even most) electronically excited states are strongly correlated. Thus, although the ground electronic state of closed-shell molecules is usually weakly correlated, the excited states tend to be strongly correlated not only in transition-metal compounds but also in organic molecules. In summary, transition metal systems with open-shell configurations, biradicals, magnetic systems, and electronically excited states are prime examples of where we may need to treat strong correlation. These examples provide strong motivation for the search for more affordable and more accurate methods of handling strongly correlated systems.

## 3. MC-PDFT and MC-NEFT

As mentioned in Section 1, although KS theory is in principle exact for all systems, it is less accurate for strongly correlated systems when used with available approximate exchange–correlation functionals. The discussion of strong correlation in Section 2 helps us understand why this is so. KS theory represents the electron density by a single Slater determinant. Strongly correlated systems are intrinsically multiconfigurational, and therefore a single Slater determinant is not a qualitatively correct wave function for the real system, even though, if one had the unknown exact KS functional, it would be adequate to get accurate results in KS theory, where the reference wave function represents a system of noninteracting electrons rather than representing the real system. In practice, we must work with approximate exchange–correlation functionals, and the exchange–correlation functional would apparently have to be very convoluted to obtain accurate results based on the noninteracting-electron starting point when it is not a qualitatively correct representation of the real wave function. Thus, it is perhaps not surprising that, although exchange–correlation functionals have − over the years − been improved for inherently multiconfigurational systems, they still do a much better job for weakly correlated systems than for strongly correlated ones.^4–6^

In an “unrestricted” KS calculation (sometimes called a spin-polarized calculation), one does not constrain the Slater determinant to be a spin eigenfunction. When KS theory is applied to strongly correlated systems, an unrestricted calculation is often required to get more accurate energetics, and the spin densities and spatial symmetry of the optimized Slater determinant often become different than that of the wave function (recall that the Slater determinant in KS theory is a reference function corresponding to a noninteracting-electron system with the same density as the actual system; it does not converge to the physical wave function as the exchange–correlation functional is improved. The KS determinant “is clearly not the correct wave function for the system.”^11^).

Consider the dissociation of H_2_ into two hydrogen atoms. If H_2_ is stretched to more than twice its equilibrium internuclear distance, the optimized Slater determinant from unrestricted KS theory no longer corresponds to a singlet state. However, the true ground state of H_2_ is a singlet at all internuclear distances, except for infinite separation where singlet and triplet states are degenerate. A singlet has the up-spin density *ρ*_α_ equal to the down-spin density *ρ*_β_ at all points in space. The wave function for significantly stretched H_2_ is a biradical with one partially bonded H atom on the left and the other on the right. A spin eigenfunction can achieve this for stretched H_2_ only by including more than one configuration. A Slater determinant can represent this only by placing an up-spin electron on the left and a down-spin one on the right (or *vice versa*), and this means that the determinant cannot possibly be a singlet; in fact, the broken-spin Slater determinant is a combination of a singlet wave function and a triplet one, and the electron spin *S* is no longer a good quantum number.

Broken symmetry is both a curse and a blessing for KS theory. It is a curse because one often obtains a Slater determinant that has the wrong spin symmetry for a multiconfigurational molecule, and that impedes interpretation of the result for some purposes. However, the blessing is that this is how KS theory predicts accurate energies. For example, for stretched H_2_, it has a hydrogen atom electron density on the left and a hydrogen atom electron density on the right, and this accurate density (even though the spin densities are wrong) can yield a useful prediction of the energy. For this reason, the spin densities in KS theory are unphysical and might better be characterized as effective spin densities to be used in the density functional but not to be confused with physical spin densities. In other words, KS theory uses unphysical spin densities (we can call them effective spin densities) to mimic the total electron density (sum of up-spin and down-spin density) that can only be properly represented by a multiconfiguration wave function. Now we return to the curse. Another aspect of the curse is that the broken-symmetry solution is not helpful when we need to have unambiguous predictions for the singlet and triplet energies, for example, if we want to calculate the spin splitting between singlet and triplet methylene or if we want to calculate properties or even the excitation energies of a particular singlet or triplet excited electronic state as a function of geometry. KS theory gives energies that correspond to unphysical reference states; in some cases, the spin splitting is a minor issue, but in other cases, the electronic spins are so mixed in the unrestricted determinant that it is not clear what state one has calculated or how to interpret its energy. A variety of nonrigorous methods have been devised to try to make accurate predictions even when this happens,^12–15^ and sometimes these methods yield useful results, but the situation is not satisfactory. The practical deficiency of KS theory due to available density functional approximations having difficulty overcoming the single-configuration representation of the density motivates using a better reference function with enough configurations to represent the physics or the density without unphysical spin mixing.

Before proceeding to MC-PDFT, it is useful to review some background on the exchange–correlation density functionals used in KS theory. KS functionals may be classified into two types. In a local functional, the energy density at a grid point depends on ingredients evaluated at that point, such as electron densities, electron pair densities, their gradients, or local kinetic energy density. In a nonlocal functional, the energy density at a grid point depends explicitly on quantities at other points in space. The most common nonlocal ingredient in a nonlocal KS functional is the Hartree–Fock (HF) exchange, which requires integration over all space (by HF exchange, we mean the exchange energy computed from a single Slater determinant as if it were the Hartree–Fock determinant. This is sometimes called “exact” exchange, but we prefer to avoid that language because when one works with multiconfigurational wave functions for real systems with interacting electrons, the exchange energy cannot be unambiguously defined for wave functions that are not single determinants. It is in this context that Gill *et al.* stated that “the exchange energy by itself is not clearly defined for densities other than Hartree–Fock.”^16^ A more detailed discussion of the issues that arise in trying to define exchange unambiguously has been given by Engel and Dreizler.^17^

The nonclassical energy functionals in MC-PDFT are called on-top functionals. The on-top functionals we used in most of our MC-PDFT work so far (the exceptions are the functionals discussed in Sections 7–9) are obtained by translating local functionals from KS theory. The resulting on-top functionals depend on the total electron density (the probability density of finding an electron at a point in space) and the on-top pair density (the probability density of finding two electrons at a point in space). We have developed two kinds of translations; one makes a functional of the density, the on-top pair density, and the gradient of the density; the other makes a functional of the density, the on-top pair density, and both of their gradients. Functionals made the former way^7^ have the prefix “t”(for “translated”), and those made the latter way^18^ have the prefix “ft” (for “fully translated”). For example, in later sections we mention tPBE and tBLYP, which are, respectively, translations of the PBE^19^ and BLYP^16^ functionals of KS theory, and we mention ftOreLYP, which is a full translation of the OreLYP^20^ functional of KS theory. The formula for a translated functional is given in Appendix A, and an explanation of the mathematics and physics behind the translation process is given in Section 6.

There is a direct connection between how energy functionals are used in KS theory and MC-PDFT. In KS calculations, one starts by forming a single-configuration wave function (in particular, a Slater determinant) from a set of orbitals, and one defines the energy as the sum of the kinetic energy and classical Coulomb energy of the single-configuration wave function plus a functional of the up-spin and down-spin densities (note that the wave function is not the exact wave function of the system under study, but using a wave function to represent the density is one way to enforce “*N*-representability”^21^). Then, one optimizes the orbitals to minimize that energy. The functional is called the exchange–correlation density functional or just the exchange–correlation functional (sometimes, one calls it the density functional, but one should be careful not to do this if the precise meaning is not clear from the context). The classical Coulomb energy is the sum of the nuclear–nuclear repulsion, the nuclear-electron attraction, and the classical formula for the electron–electron repulsion; it may also be called the classical electrostatic energy. We label the sum of the kinetic energy and classical Coulomb energy as the classical energy and the remainder as the nonclassical energy; in this sense, an exchange–correlation functional is a nonclassical-energy functional.

In MC-PDFT, one uses orbitals to form a multiconfiguration wave function, usually a multiconfiguration self-consistent-field (MCSCF) wave function. In an MCSCF wave function, the orbitals and the coefficients of the CSFs are optimized to minimize the expectation value of the electronic Hamiltonian; the MC-PDFT energy is then defined as the sum of the kinetic energy and classical Coulomb energy of the multiconfiguration wave function plus a functional of the electron density and the on-top pair density:3.1
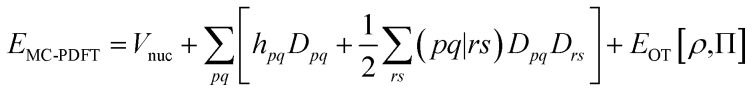
where *V*_nuc_ is the nuclear repulsion, *p*,*q*,… are indices of general orbitals, *D*_*pq*_ is an element of the one-electron reduced density matrix (1-RDM), *h*_*pq*_ is a matrix element of the electronic kinetic energy plus the electron–nuclear attraction, (*pq*|*rs*) is a two-electron integral in the Mulliken notation, and Π is the on-top pair density (note that the MCSCF wave function is not the exact wave function of the system under study). The functional *E*_OT_[*ρ*,Π] is called the on-top functional, and a goal of the theoretical development is to define expressions for the on-top functional such that eqn (3.1) gives an accurate electronic energy. The sum of *V*_nuc_ and the summation term in the equation is the classical energy, and the final term is the nonclassical energy, as calculated by the on-top functional. Therefore, we can again use the term nonclassical-energy functional. In most of our MC-NEFT work so far, the nonclassical-energy functional is the on-top functional, yielding MC-PDFT. More generally, one can replace *E*_OT_[*ρ*,Π] by a nonclassical-energy functional that depends on other ingredients, such as the density coherence or neural network input features. MC-PDFT is a special case of MC-NEFT. Further mathematical details of basic MC-PDFT are reviewed in Appendix A, and the definition and development of pair-density functionals and more general nonclassical energy functionals are presented in Sections 6–9.

There is a major difference between the strategy summarized in the previous paragraph and the many other ways multiconfiguration wave functions have been combined with density functional theory in the literature. In most other approaches, one calculates the energy of the multiconfiguration wave function in the usual way (*i.e.*, as the expectation value of the Hamiltonian) and then adds on a density functional “correction” for the remaining correlation energy. However, these approaches suffer from the difficulty that there is no clear way to translate the amount of correlation already present in the expectation value into density functional language so that one can safely add in only the correlation energy that is not already included. This is called the double-counting problem. This kind of potential double counting, where there is correlation energy in both the energy of the multiconfiguration wave function and the correlation functional, does not affect MC-PDFT because we do not use the energy of the multiconfiguration wave function; rather we calculate the energy as the sum of a kinetic energy term, a classical Coulomb energy, and an on-top energy. There is another way to avoid double counting, namely, to use range separation and − for example − to calculate the Coulomb energy arising from short-electron-electron distances by density functional theory and the part arising from long interelectronic distances by wave function theory.^22,23^ To date, we have not combined that approach with MC-NEFT, although a method with this combination has been reported by Hapka *et al.*,^24^ who found that the method “provides accuracy comparable with more computationally expensive *ab initio* rivals.” The λ-DFVB valence-bond-based multireference density functional method avoids double counting by decomposing the electron–electron interactions into a wave function term and a density functional term with a variable parameter *λ*.^25^ Yet another way to blend wave function theory with density functional theory is hybridization, discussed in Section 7.

It is sometimes suggested that the possibility of double counting in methods combining wave function theory and density functional theory should be discussed in terms of the correlation kinetic energy. We consider the treatment of the kinetic energy further in Appendix C.

As mentioned above, the single-configuration wave function used to represent the density in KS theory is a Slater determinant, which is an antisymmetrized product of spin–orbitals. A simple product wave function has no correlation between spin–orbitals, but the antisymmetrization prevents two same-spin electrons from being at the same point. Opposite-spin electrons remain uncorrelated. For a closed-shell singlet with doubly occupied orbitals, the up-spin density equals *ρ*(**r**)/2, and the down-spin density equals *ρ*(**r**)/2, where *ρ*(**r**) is the total density at **r**. Since the opposite-spin electrons are uncorrelated, the probability of finding an up-spin and a down-spin electron at point **r** is, therefore, equal to [*ρ*(**r**)/2]^2^. For an open-shell system or a multiconfigurational wave function of a closed-shell system, the on-top pair density differs from [*ρ*(**r**)/2]^2^; this deviation contains information about the character of the multiconfiguration wave function, and MC-PDFT uses this information as one of the inputs to the on-top functional. In our recent work generalizing MC-PDFT to MC-NEFT, we have shown that we can use other ingredients besides density and on-top pair density in the energy functional. For example, we can use the density coherence instead of the on-top pair density.^26^ The development of multiconfiguration density coherence functional theory is discussed in Section 8.

We now turn our attention to the wave function component of MC-PDFT. Although, in principle, PDFT may be combined with any wave function method or even density matrix methods,^27,28^ the most explored direction so far for surpassing the capabilities of KS theory comes from combining PDFT with explicitly multiconfigurational wave functions, mainly MCSCF. The most popular version of MCSCF is complete active space SCF,^29^ abbreviated CASSCF. In this method, the wave function includes all CSFs formed by placing *m* active electrons in all possible ways in all *n* active orbitals, with the remaining electrons in doubly occupied orbitals (called inactive orbitals) in all CSFs. The user-defined active space is often abbreviated as (*m*,*n*). A CI calculation that includes all possible CSFs that one can form with a given basis set is called full CI (FCI); therefore, CASSCF can be thought of as the FCI calculation within the delimited space of active electrons and orbitals combined with a Hartree–Fock treatment of the inactive orbitals. To date, CASSCF has been the most common reference wave function for MC-PDFT, sometimes referred to as CAS-PDFT. Sections 4 and 5 discuss using other choices of ground state MCSCF wave functions. In Sections 7 and 10, we discuss state-averaged CASSCF (SA-CASSCF) reference wave functions, in which the orbitals and CI coefficients are optimized to minimize the average of the energies of several low-energy states, which is in contrast to the original CASSCF wave function that is optimized to minimize the energy of only the ground state. The SA-CASSCF method is useful for the description of excited states and for describing the ground state well when it is closely coupled to a low-lying excited state. Section 4 will discuss other reference functions for MC-PDFT.

We have seen that MC-PDFT is a blended method. It starts with a reference wave function to get the kinetic energy, the density, and on-top pair density. Then these functions are used as inputs to an on-top functional to obtain the total energy. The remainder of this perspective will focus on both of these important issues, which represent the pillars of MC-PDFT development: (i) getting a good multiconfiguration reference function, and (ii) getting a good nonclassical-energy functional. And we will also cover more general forms of MC-NEFT.

## 4. LAS-PDFT, GAS-PDFT, and SP-PDFT

The number of configurations in a CASSCF calculation increases exponentially with respect to the size of the active space because of the large number of CSFs included in the complete active space.^30^ For a given (*m*,*n*), one can decrease the number of CSFs by using restricted active space SCF (RASSCF)^31,32^ or generalized active space SCF (GASSCF),^33^ which involve partitioning of the active space into subspaces with restricted excitations. While these approaches reduce the CSF space, computational cost still grows rapidly with system size. Therefore, it is difficult to obtain suitable multiconfiguration reference wave functions for MC-PDFT calculations on large systems. Another way to use partitioning to decrease the cost is localized active space SCF (LASSCF),^34,35^ whose use as the reference function for MC-PDFT yields LAS-PDFT.^36^

**Fig. 1:**
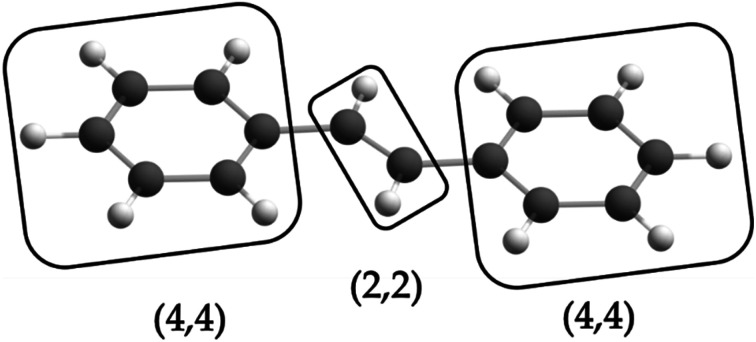
One possible fragmentation of the complete active space of the stilbene molecule, which corresponds to a 200-fold reduction in the number of CSF coefficients of the LAS wave function compared to the CAS wave function.

LASSCF factorizes a wave function within the active space into separate localized parts, so that the overall wave function is4.1
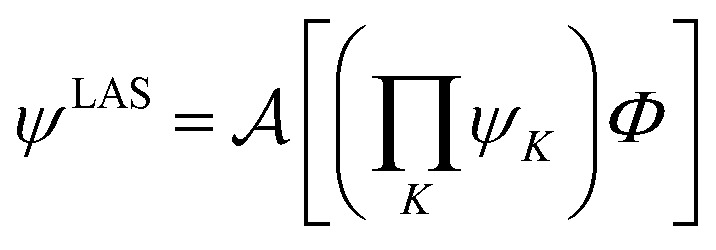
where *ψ*_*K*_ is a general many-body wave function defined in a localized subset of active orbitals, *Φ* is the single determinant of inactive orbitals, and 
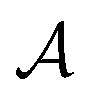
 is the antisymmetrizer. For instance, in the stilbene molecule, an overall (10,10) active space can be decomposed into two (4,4) active subspaces localized on each phenyl ring and one (2,2) active subspace localized on the linking ethylene unit (see Fig. 1). Making this approximation reduces the number of CSFs by a factor of 200, and the ratio is even greater with larger active spaces.

**Fig. 2:**
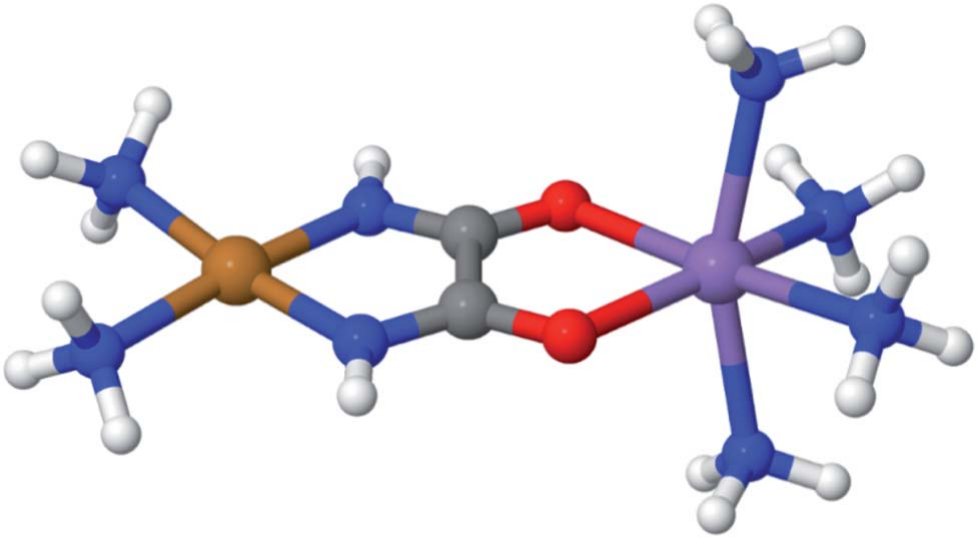
A bimetallic complex ([Cu(NH_3_)_2_]oxamide[Mn(NH_3_)_4_]^2+^)whose spin-state energy gaps were explored in ref. 36.

LASSCF is physically motivated by the observation that static electron correlation is often localized on distinct subunits, and it is most useful when that is the case. Strong correlation between subsystems requires one to go beyond combining subsystem wave functions by multiplying them together and antisymmetrizing the product. As an example of how this works out in practice, consider the unsaturated d-orbital subshells of bimetallic transition metal complexes such as the one depicted in Fig. 2. Clearly, near-degeneracy effects on each metallic center lead to a high multiconfigurational character. However, the d-shell electrons around two separate transition metal ions are separated by several Å, and for many purposes, that will mean that they can be treated as interacting with one another only in a mean-field way. This conjecture is confirmed by the results of CAS-PDFT and LAS-PDFT calculations on the *T*_1_ excitation energy carried out in ref. 36 using a minimal (6,6) complete active space: CAS-PDFT predicts 42.3 kcal mol^−1^, and LAS-PDFT predicts 43.1 kcal mol^−1^. In situations such as this, LAS-PDFT is a computationally more affordable route to CAS-PDFT-quality results.

We can interpret a comparison of LAS-PDFT to CAS-PDFT as a probe of the validity of the approximation underlying LASSCF. When a relative energy computed by LAS-PDFT agrees much more closely with CAS-PDFT than the reference LASSCF wave function agrees with the reference CASSCF wave function, then we infer that electron correlation between fragments that is missing from the former is indeed “weak” or “dynamic”. However, if LAS-PDFT and CAS-PDFT disagree, we can infer that the factorization of the active-space wave function was less well justified.

**Fig. 3:**
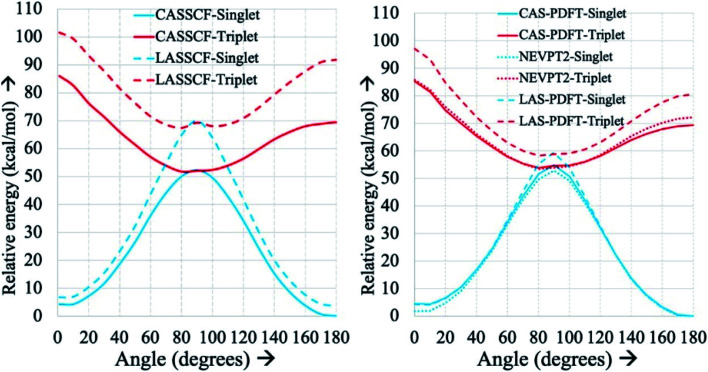
Relaxed ground-state singlet and triplet potential energy curves for the *cis*–*trans* isomerization of stilbene, computed by various methods with an overall (10,10) active space and a 6-31G basis set, relative to the CASSCF (left) or CAS-PDFT (right) singlet energy of *trans*-stilbene. Reproduced with permission from ref. 36.

Fig. 3 displays an example of this analysis for the ground-state singlet and triplet relaxed potential energy curves for the *cis*–*trans* isomerization of the stilbene example mentioned above.^36^ LAS-PDFT and CAS-PDFT are everywhere in closer agreement than LASSCF and CASSCF for the energy difference between the *cis* and *trans* isomers. This agreement suggests that the LASSCF reference wave function is a reasonable zero-order approximation.

However, compared to CAS-PDFT and second-order N-electron valence perturbation theory (NEVPT2), LAS-PDFT overestimates the relative energy of the singlet state near the transition structure (and the triplet state everywhere) by 5–10 kcal mol^−1^. This is because this three-fragment LASSCF wave function is qualitatively less correct for open-shell states of stilbene than for closed-shell states. For both the singlet state at the transition geometry and the triplet state, LASSCF must localize the diradical character of the electronic state on a single fragment, while the singlet reactant and product are well described by closed-shell wave functions. The more qualitatively correct picture provided by CASSCF, is that the unpaired electrons are delocalized across the phenyl rings and the ethylene linker. Although the PDFT energy includes dynamic correlation effects missing from LASSCF or CASSCF, it cannot fully overcome a qualitatively incorrect reference wave function.

The above discussion shows that LASSCF is not appropriate for every problem, but when it is appropriate, it provides a way to extend MC-PDFT to larger systems, even systems with multiple sites and significant static correlation, provided the strong correlation is entirely intra-site.

A step beyond LASSCF is GASSCF. The difference between LASSCF and GASSCF can be illustrated by considering a system composed of two fragments (A and B), whose active electrons and active orbitals form subspaces of the active space of the entire molecule. Fragment CASSCF wave functions would have the form:4.2
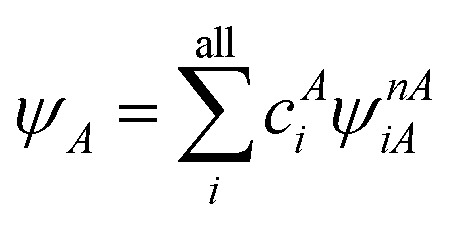
4.3
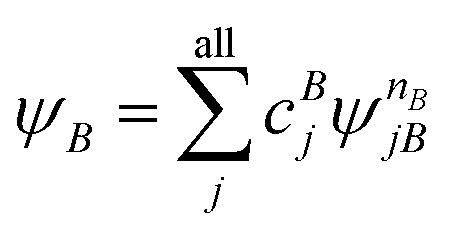
where 
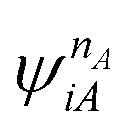
 and 
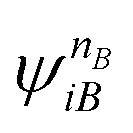
 are fragment CSFs describing *n*_*A*_ and *n*_*B*_ electrons, respectively. A CASSCF wave function on the entire system would have the form4.4
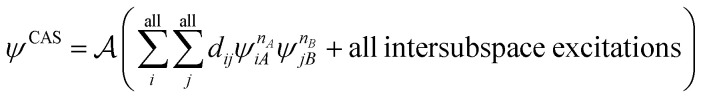
where the orbitals and CI coefficients are re-optimized. A LASSCF wave function would have the form4.5
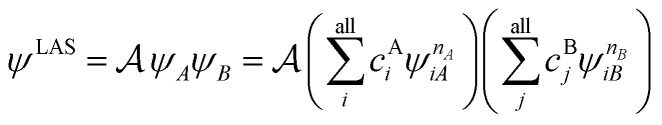
with the orbitals and CI coefficients optimized for this form. A GASSCF calculation without intersubspace excitations would have the form4.6
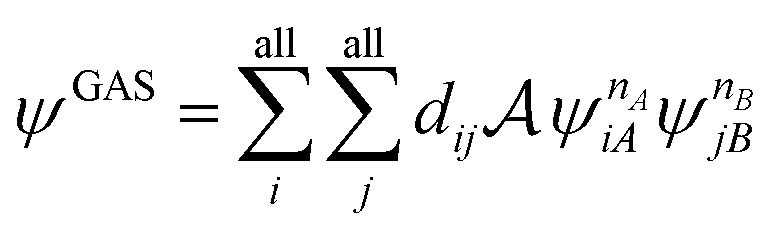
with the orbitals and *d*_*ij*_ coefficients optimized for this form. The GASSCF wave function is more compact than the CASSCF one because the interspace excitations are missing (thereby eliminating potential “deadwood” in the configuration space), but it is more complete than a LASSCF wave function because the subspaces are coupled beyond the mean-field level, thereby allowing one to treat strong correlation between the subspaces. The extension of GASSCF to a larger number of subspaces and to subspaces defined more generally (not defined in terms of fragments) is straightforward.

**Fig. 4:**
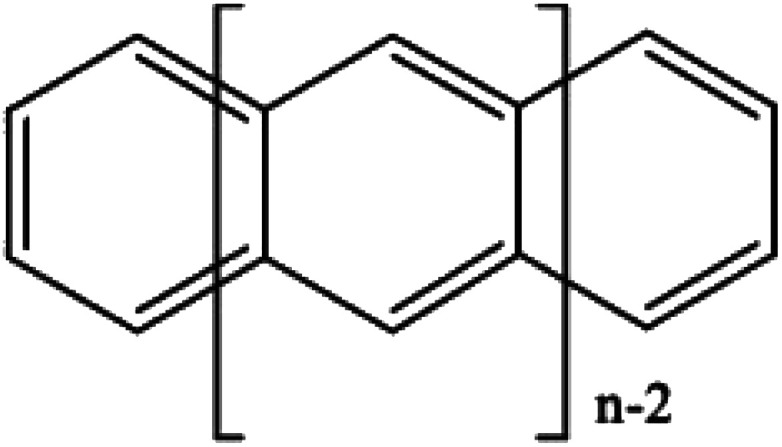
A linear polyacene with *n* rings.

If desired, one could allow a limited number of interspace excitations in GASSCF. An illustrative example includes the singlet–triplet excitation energies of linear polyacenes.^37^ The structure of a linear polyacene with *n* rings is shown in Fig. 4. The number of π orbitals in each structure is 4*n* + 2, with only half occupied in the dominant singlet CSF. We used an active space containing all π orbitals and electrons. We considered various ways to partition the active space, and here we discuss the frontier partitioning scheme. In this scheme, we first made three subspaces. One (called GAS2) has 2 electrons in two orbitals, the HOMO and LUMO; another (called GAS1) has the remaining 2*n* occupied π orbitals, and the final one (called GAS3) has the remaining 2*n* unoccupied π orbitals. We allowed single and double intersubspace excitations from GAS1 to GAS3. For *n* = 8, the CAS singlet wave function has ∼10^17^ CSFs, whereas the frontier-partitioned GAS triplet wave function has 37 000. For *n* = 12, the CAS singlet wave function has ∼10^27^ CSFs, and the GAS one has only 190 000. Averaging over eleven cases (*n* = 2–12, corresponding to active spaces ranging from (10,10) to (50,50)), the mean deviation of the GAS-PDFT vertical excitation energy from the best available estimate is 1.5 kcal mol^−1^, as compared to 4.9 kcal mol^−1^ for the results calculated as Hamiltonian expectation values with the reference GASSCF wave functions.

We have also pursued a systematic method for using GAS partitioning without intersubspace excitation. In this method, each subspace contains only one or two orbitals and only one, two, or three electrons. This scheme is called the separated pair (SP) approximation^38^ and it was included in our previous review;^9^ therefore, we limit the discussion here to a statistical analysis, published^39^ after that review, of our applications of the SP approximation to 25 diatomic molecules that contain one or two transition metal atoms and for which accurate bond dissociation energies are available^39–42^ (for two of the cases, we use extended SP theory rather than SP theory; in extended SP theory, one puts all singly occupied orbitals and their correlating orbitals into a single subspace^42^). In each case we also carried out CAS-PDFT calculations for comparison. The 25 molecules are CrH, MnH, FeH, CoH, FeC, ScN, VN, CrN, TiO, FeO, NiO, V_2_, Cr_2_, CrF, NiC, FeS, NiS, FeSe, NiSe, TiS, TiC, WCl, VSi, NbSi, and TaSi. We use nominal correlated-participating-orbitals^43,44^ (*nom*-CPO) active spaces. The mean unsigned errors in the SP-PDFT bond dissociation energies with the ftOreLYP functional for these 25 highly multireference molecules is only 0.21 eV. And yet the SP reference wave functions are quite compact. For example, for the ground state of TaSi, the *nom*-CPO CASSCF reference wave function has 2320 CSFs, whereas the *nom*-CPO SP reference wave function has only 272 CSFs.^39^

The success of the SP and extended SP approximations is particularly encouraging in light of a recommendation we made in a paper devoted to the study of active space dependence, where we wrote^45^ “This leads to the recommendation that, at least with the presently available on-top density functionals, MC-PDFT should be employed with a small active space, large enough to account for near-degeneracy correlation but not so large as to include significantly more dynamic correlation”. The SP and extended SP methods can be considered an attempt to systematically define an active space that is large enough to account for near-degeneracy correlation in reactions involving bond breaking but is not significantly larger (although one expects a problem for very large active spaces, unless one derives new density functionals appropriate for them, in practice we have found that the error does not depend strongly on the precise constitution of the active space for alternative reasonable choices of the active space).

## 5. DMRG-PDFT

Another way to make MCSCF wave function calculations on large systems practical is to use the density matrix renormalization group^46–51^ (DMRG). The DMRG algorithm is a powerful and efficient way to approximate FCI for large active spaces, and it can replace a conventional FCI algorithm in the CI portion of a CASSCF calculation. In DMRG, the wave function is approximated by a matrix product state (MPS),^52,53^ and the number of basis states employed to represent this MPS is limited by a parameter called the bond dimension. If the MPS were not limited by the bond dimension, the DMRG wave function would be equivalent to FCI. The bond dimension, which is usually denoted as *M*, should in principle be increased until the results are converged; it happens that this convergence is typically achieved quickly enough that the number of independent parameters specifying the DMRG wave function is much less than the number of configurations in an FCI wave function, and therefore the cost of the calculation is much less expensive than CASSCF with a conventional FCI solver. This enables the application of DMRG-SCF (MCSCF with DMRG approximation in the CI portion of the calculation) to larger active spaces than those multiconfigurational methods with conventional algorithms.

Even though a DMRG-SCF wave function corresponds to a larger active space, it is still quantitatively inaccurate (even for the largest affordable active spaces) because it contains an insufficient fraction of the dynamic correlation (*i.e.*, it only approximates large-active-space CASSCF, and CASSCF is slowly convergent with respect to increasing the size of the active space). To include dynamic correlation fully, the DMRG-SCF wave function can be used as the reference function for multireference CI^54,55^ (MRCI), second-order complete active space perturbation theory^49^ (CASPT2), NEVPT2,^56^ or other multireference methods. However, four-particle reduced density matrices (RDMs) are needed for DMRG-MRCISD, DMRG-CASPT2, and DMRG-NEVPT2, and even higher order of RDMs are needed to include higher levels of dynamic correlation in these methods. The four-particle RDMs can be approximated by the DMRG-cu(4)-MRCI^54^ and DMRG-cu(4)-CASPT2 (ref. 57) methods, in which the highest-order of RDMs needed are three-particle RDMs, and this provides one way to reduce cost. A more economical way to approximate full dynamic correlation with a DMRG-SCF reference function is to use a DMRG-SCF wave function as a reference function for MC-PDFT; when this is done, the resulting DMRG-PDFT calculations need no more than two-particle RDMs. Since the two-particle RDMs are already available in the DMRG-SCF calculation, extra calculations for higher orders of RDMs are avoided in the DMRG-PDFT method. Therefore, combining DMRG-SCF and MC-PDFT provides an efficient way to approximate the full dynamic correlation with a DMRG-SCF reference function at an affordable computational cost.

The DMRG-PDFT method has been applied in various electronic structure computations.^58–61^ The application of DMRG-PDFT to the singlet-triplet gap in polyacenes and polyacetylenes gave results close to experimental values and high-level wave function calculations. DMRG-PDFT was also applied to the ligand-free iron porphyrin molecule in a (34,35) active space to predict the energetic ordering of the singlet, triplet, and quintet spin states. The DMRG-PDFT calculations on the polyacenes, polyacetylenes, and iron porphyrin molecules showed a rapid convergence of the energy with respect to the bond dimension. The magnetic coupling in a tris-hydroxo-bridged chromium dimer is an example of how DMRG-PDFT can be applied to a magnetic property calculation, and the results given by the DMRG-PDFT method are in good agreement with experiments.

In the magnetic property application,^60^ we calculated spin-gaps and magnetic coupling constants in a tris-hydroxo-bridged chromium dimer. A CASSCF calculation with an active space of only the Cr d orbitals is insufficient to describe the system well, so we used DMRG-PDFT with a (30,22) active space and a bond dimension of 1000, yielding a magnetic coupling constant of −68 cm^−1^, in good agreement with an experimental value^62^ of −66 cm^−1^. In contrast, DMRG-SCF gave −22 cm^−1^, which showed that the external correlation energy is very important even for a large active space. Because our active space was large enough to include ligand orbitals, we were able to use the DMRG-PDFT unpaired electron density to show that a significant magnetic coupling occurs through ligand-mediated superexchange in conjunction with through-space coupling; this is a more direct way to demonstrate superexchange than the previous work in the literature.

DMRG-PDFT is also available in the Fourier-multistate-PDFT (FMS-PDFT), which is a multistate PDFT (MS-PDFT) method (MS-PDFT methods are discussed in Section 10). Overall, the high accuracy and low computational cost (almost negligible compared with DMRG-SCF) show the DMRG-PDFT method has great promise for electronic structure calculations on complex molecular systems.

## 6. On-top functionals

The process of translation and full translation to obtain on-top density functionals is explained in the original articles^7,63^ and a formula is given in Appendix A. The present section gives an explanation that may serve as an introduction to the thinking that motivates the translation. This section is written for the case where spin–orbit coupling is neglected, which is a good approximation for light atoms. When spin–orbit coupling is neglected the exact electronic wave function is always a spin eigenfunction, by which we mean an eigenfunction of *S*^2^, where *S* is total electron spin. Approximate wave functions that are spin eigenfunctions are said to maintain spin symmetry.

It is useful, as background, to start with Hartree–Fock theory, which is a wave-function-based variational method that represents the wave function as a single Slater determinant. Hartree–Fock theory cannot represent the true wave function well in strongly correlated systems, and if one solves the Hartree–Fock equations for strongly correlated systems with the constraint that the Slater determinant is a spin eigenfunction, one often gets a very inaccurate energy (for example, the error in the dissociation energy of singlet H_2_ is ∼6 eV, which is larger than the bond energy itself, and which yields a qualitatively incorrect potential energy curve). However, if one relaxes the constraint that the Slater determinant is a spin eigenfunction and simply finds the variationally best energy for each internuclear distance,^64^ one finds a much more accurate bond energy and a qualitatively correct potential energy curve. Solving the Hartree–Fock equations by finding the variationally best solution without the restriction that the Slater determinant be a spin eigenfunction is usually called a spin-unrestricted calculation or simply an unrestricted calculation. The solution is sometimes called a broken-symmetry solution. The key point to be made for the present purposes is that allowing the spin densities to be incorrect allows the total densities (sums of up-spin and down-spin densities) to better approximate the true total densities within the limitation of a single Slater determinant, and calculating the variational energy from the broken-symmetry solution with these better densities gives a more accurate energy than is obtained when constraining the Slater determinant to be a spin eigenfunction.

A configuration interaction calculation involves a linear combination of CSFs with various orbital occupancies, and using multiple CSFs can introduce unpaired electrons into an approximate wave function while maintaining spin symmetry,^65^ but a Slater determinant cannot do this. A single Slater determinant has a single set of orbital occupancies, and we interpret the incorrect up-spin and down-spin densities of the unrestricted calculations as a way to introduce unpaired electrons into the solution when one has only a single set of orbital occupancies.

Kohn–Sham theory uses the Slater determinant in a different way. If one were able to use the exact exchange–correlation functional (which cannot be used because it is unknown for a general system), the single-particle probability density of the Kohn–Sham Slater determinant would be the same as the true single-particle probability density of the real system, although the Slater determinant would not be the same as the wave function of the real system (in practice, one has an approximate exchange–correlation functional, and the single-particle probability density of the resulting Slater determinant is only an approximation to the true single-particle probability density). Discussing the spin properties of the solution to the Kohn–Sham equations with the unknown exact functional is complicated by the fact that two options exist for how to treat spin; in either option, though, the Slater determinant need not have the same expectation value of *S*^2^ as does the real wave function.^66^ In this regard it is relevant to recall that *S*^2^ is a two-electron property whose value depends on the two-electron reduced density matrix. Although the energy is a functional of the two-electron reduced density matrix, and Kohn–Sham theory with the exact functional would give the correct energy, it does not compute the energy from the two-electron reduced density matrix, and it does not give correct two-electron reduced density matrix. It is a remarkable feature of KS theory that the unknown exact functional would give the correct potential energy curve even when the Slater determinant cannot provide an even qualitatively correct treatment of the exact wave function.

In practice, though, we do not have the exact density functional for either Kohn–Sham option, and one simply has a choice in practical work of whether or not to require the Slater determinant to be a spin eigenfunction. With the available approximate density functionals, the situation is remarkably similar to the situation with Hartree–Fock theory, that is, if one solves the Kohn–Sham equations for strongly correlated systems with the constraint that the Slater determinant is a spin eigenfunction, one often gets a very inaccurate result. However, if one relaxes the constraint the Slater determinant is a spin eigenfunction and simply finds the variationally best solution (for the given approximate density functional) for each internuclear distance, the total energies tend to be more accurate. Solving the Kohn–Sham equations by finding the variationally best solution without the restriction that the Slater determinant be a spin eigenfunction is usually called a spin-unrestricted calculation or simply an unrestricted calculation by chemists and is called a spin-polarized calculation by physicists. The solution is sometimes called a broken-symmetry solution. Just as in the Hartree–Fock case discussed above, allowing the spin densities to be incorrect allows the total densities to better approximate the true total densities (sums of up-spin and down-spin densities) within the limitation of a single Slater determinant, and calculating the energy from the broken-symmetry solution gives a more accurate energy than is obtained when constraining the Slater determinant to be a spin eigenfunction. We interpret the incorrect up-spin and down-spin densities of the unrestricted calculations as a way to introduce unpaired electrons into the solution when one has only a single set of orbital occupancies. To emphasize that the spin densities are not physical when using the unrestricted theory, we may call them effective spin densities. The key point is that the available approximate Kohn–Sham density functionals work by using these effective spin densities to distinguish the energetic effects of paired and unpaired electrons.

MC-PDFT uses an MCSCF reference wave function rather than a single Slater determinant, and the MCSCF wave function is a spin eigenfunction with physical spin densities. Therefore, since currently available approximate density functionals use unphysical effective spin densities to mimic the effects of unpaired electrons and do not give accurate energies for strongly correlated systems when physical spin densities (such as the spin densities of MCSCF wave functions) are used, MC-PDFT would be inaccurate if it used the MCSCF spin densities with currently available Kohn–Sham density functionals. We have two alternatives: (i) we can devise new kinds of density functionals for MC-PDFT based on new kinds of considerations, or (ii) we can extract effective spin densities from MCSCF wave functions that will allow us to use Kohn–Sham density functionals. Although alternative (i) is an interesting challenge, and is worth pursuing, we have originally chosen to use alternative (ii), which we call “translation” and which we discuss next.

Our translation process uses not the spin densities of the MCSCF wave function, but rather the total density (sum of the spin densities) and the on-top pair density. Whereas the density *ρ*(**r**) is the probability density of finding an electron at a point **r** in space, the on-top pair density Π(**r**) is the probability density of finding two electrons at a point **r** in space. For Slater determinants, the magnetization spin density is given by^67^6.1
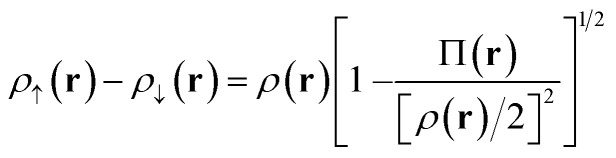


Note that, by the Pauli principle, two electrons can be at the same point in space only if they have opposite spins. For a closed-shell Slater determinant, since *ρ*_↑_(**r**) is the same as *ρ*_↓_(**r**) [both equal *ρ*(**r**)/2] and since the electronic motions are uncorrelated in a Slater determinant except for the correlation imposed by antisymmetry, the on-top pair density is simply [*ρ*(**r**)/2]^2^; then the bracketed quantity vanishes in eqn (6.1). Thus, by eqn (6.1), the magnetization spin density vanishes everywhere in space for a closed-shell singlet, as it should for any singlet. But the bracketed quantity in eqn (6.1) does not normally vanish for a multiconfigurational singlet wave function; in fact, it provides effective spin densities that differ from the true spin densities and actually mimic the unrestricted Kohn–Sham effective spin densities. Thus eqn (6.1) is used to provide the starting point for our translation.

For Slater determinant, the bracketed quantity in eqn (6.1) is greater than or equal to zero, but for a multiconfiguration wave function it can be negative. To avoid the unphysical negative values, we obtain effective spin densities by6.2
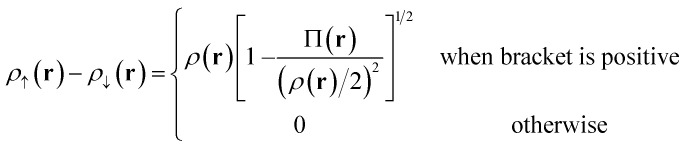
and

6.3 *ρ*_↑_(**r**) + *ρ*_↓_(**r**) = *ρ*(**r**)

This would define the on-top density functional if the Kohn–Sham functional depended only on the spin densities. However, we apply this also to Kohn–Sham functionals that are gradient approximations, *i.e.*, functionals that also depend on the magnitudes of the gradients of the spin densities. In our “translated” protocol we obtain effective spin density gradients from eqn (6.2) and (6.3) by neglecting the gradient of Π(**r**), yielding the formula in Appendix A. In our “fully translated” protocol, we include the gradient of Π(**r**), and we use a smoothing function to make the transition from the positive-bracket region to the negative-bracket region continuous through the second derivative. This yields more cumbersome expression^63^ that need not be reproduced here.

## 7. Hybrid MC-PDFT

We have reviewed above how MC-PDFT blends the multiconfigurational (MC) wave function theory (WFT) and density functional theory (DFT), taking advantage of the attractive features from both. From WFT it inherits the physically sound and qualitatively correct description of the multiconfigurational wave function, and from DFT it inherits the ability (at least in principle) to include all-electron correlation energy by an affordable nonclassical-energy functional. MC-PDFT, however, also inherits some inadequacies of both WFT and DFT. From WFT it inherits the difficulty of deciding on a reference wave function and the possibility that any affordable reference wave function will not be good enough. The DFT side inherits the difficulty of developing an accurate functional. The latter difficulty is compounded in MC-PDFT because we use the functional with a variety of reference wave functions, whereas in KS theory, the reference wave function is always a single Slater determinant. The present section introduces the recently developed hybrid MC-PDFT, which may be viewed as an analog of hybrid Kohn–Sham theory.

Two widely recognized types of error in KS theory are delocalization error and self-interaction error. These two kinds of error are closely related in that it is the self-interaction that tends to make KS densities too delocalized; the delocalization occurs because the interaction of an electron with itself in the energy expression used to optimize KS orbitals causes the orbitals to spread out when they are optimized using that energy expression. It has been observed that MC-PDFT, which optimizes the orbitals with an energy expression that has no self-interaction, does not suffer from delocalization error.^68^ Nevertheless, there is some self-interaction error (SIE) in the MC-PDFT energy when it is calculated with a local functional. This is because a local energy density cannot fully represent the effect of electron exchange, which is intrinsically nonlocal.^69^ Historically, one of the major steps forward in improving the accuracy of KS theory was the introduction of nonlocal HF exchange into the density functional; this introduction yields what is called hybrid KS theory (also called generalized Kohn–Sham theory^70^).

We have recently developed hybrid MC-PDFT (HMC-PDFT) in which the nonclassical energy contains a nonlocal ingredient. Before explaining HMC-PDFT, we mention some related previous work. Sharkas *et al.* proposed a one-parameter hybrid density functional theory (MC1H) that uses a single parameter to interpolate between DFT and multiconfiguration wave function energies.^71–74^ This approach is also followed in pair coupled-cluster doubles λ-DFT (pCCD-λDFT)^75^ and λ-density functional valence bond (λ-DFVB).^76^ More recently, a hybrid MC-PDFT was suggested by Mostafanejad *et al.*^77^ They use scaling relations of the exchange and correlation energies with respect to the density to argue that the coefficient *λ*_c_ scaling the density functional correlation contribution needs to be the square of the coefficient *λ*_x_ scaling the for density functional exchange. In our method, which is discussed next, and which is called hybrid MC-PDFT or HMC-PDFT,^78^ the same coefficient *λ* is used for exchange and correlation.

Our HMC-PDFT method is analogous to Becke's^79^ original hybrid of Hartree–Fock theory with KS theory. In Becke's method, the hybrid KS energy is defined as a simple linear combination of the wave function and density functional energies. In the same vein, the HMC-PDFT energy is given by:

7.1 *E*_HMC-PDFT_ = *λE*_MCSCF_ + (1 − *λ*)*E*_MC-PDFT_

where *λ* is the hybridization parameter.

To compare this with the method of Mostafanejad *et al.*, we also proposed a generalization of this formulation in the form of 2HMC-PDFT that has two semi-empirical parameters: the hybridization fraction *λ* and the exponent of correlation energy *k*. The 2HMC-PDFT energy is defined as:

7.2 *E*_2HMC-PDFT_ = *E*_MC,class_ + *λE*_MC,XC_ + (1 − *λ*)*E*_X_ + (1 − *λ*^*k*^)*E*_C_

where *E*_MC,class_ is classical energy of the multiconfiguration wave function, *E*_MC,XC_ is electron correlation energy from the multiconfiguration wave function, and *E*_X_ and *E*_C_ are respectively the exchange and correlation portions of the on-top density functional energy. The 2HMC-PDFT with *k* = 2 corresponds to the global-hybrid method proposed by Mostafanejad *et al.* while *k* = 1 corresponds to HMC-PDFT.

One advantage of using the same parameter for exchange and correlation is that the method could be applied using nonseparable gradient approximations in which one approximates the sum of exchange and correlation without approximating them separately.^80,81^

Our investigations suggest that the HMC-PDFT has the same accuracy as the double-parameterized 2HMC-PDFT method. Most test sets show little to no dependence on the exponent *k*, and even when dependence is notable, the accuracy of the results is primarily decided by the value of *λ* and not *k*. We also have reported the HMC-PDFT energies for the tPBE and tBLYP functionals that show minimal dependence on the choice of functional for both the optimal value of *λ* as well as the mean unsigned errors (MUEs) at that value of *λ* across all the datasets explored in ref. 78. Mostafanejad *et al.* also report that the performance of their global-hybrid functional is equally good for a wide variety of functionals.^77^

In practice, for the cases we studied so far, there is almost no change in the optimal value of *λ* with systematic increases in the size of the active space.^78^ Tests on spin-flip atomic excitations show that CASSCF, MC-PDFT, and HMC-PDFT) all lead to a lower MUE with increasing size of the active space. For smaller active spaces, MC-PDFT is more accurate than CASSCF, and for the larger active spaces it is the other way around for spin splittings. But, for both active spaces studied, not only does HMC-PDFT perform better than the other methods, but it does so at nearly the same (50–55%) hybridization. Similar behavior is seen for the singlet and triplet excitations in benzene, where CASSCF and MC-PDFT give incorrect ordering with the smaller active space and only qualitatively accurate results for the larger active spaces, but HMC-PDFT (at 40–50%) gives quantitatively accurate results with all active spaces. This occurs even for the (6,6) active space, which does not include the important ionic CSFs.^82^ The tests on a larger set of spin-flip and spin-conserving excitations in organic molecules showed that HMC-PDFT with roughly 25% hybridization performs the best, and the conclusions from this larger dataset may be generally applicable. A larger and more diverse set of excitations with a greater variety of active space sizes is needed to determine the optimal *λ* more broadly since, in principle, it can be dependent on the nature of the excitation and the quality of the active space.

We know that the inclusion of HF exchange in hybrid KS theory sometimes increases static correlation error;^3^ however, in HMC-PDFT, the orbitals and the electron density are not affected by *λ*, and we do not expect this problem to occur in HMC-PDFT. Another difference of HMC-PDFT from hybrid KS theory is that hybrid KS theory is more expensive than local-functional KS theory, but HMC-PDFT has the same cost as MC-PDFT.

In KS theory, the hybrid functional formed by replacing 25% local exchange with HF exchange is considered as a good standard choice and is called PBE0.^83^ We made an analogous functional for HMC-PDFT with *λ* = 0.25 and with tPBE as the functional in second term of eqn (7.1); we call this tPBE0. In all cases studied (which includes spin-changing excitations, spin-conserving excitations, and bond energies), HMC-PDFT with tPBE0 had a lower MUE than either CASSCF or MC-PDFT with tPBE. Although 0.25 is not necessarily the best value of *λ* for all cases, it is very encouraging to see the ability of HMC-PDFT with a single standard value of *λ* to provide good results for multiple properties.

## 8. Multiconfiguration density coherence functional theory

So far, all MC-NEFT methods discussed in this perspective use the density and on-top pair density, which are respectively the diagonal elements of the 1-RDM and the 2-RDM in the coordinate representation. The 1-RDM, on the other hand, is simpler than the 2-RDM.^84^ The off-diagonal elements of the 1-RDM, which are called the density coherence,^85,86^ are not used as ingredients of the energy functionals of MC-PDFT^7^ and local KS theory functionals^87,88^ (although HMC-PDFT,^78^ HF exchange terms in hybrid KS theory,^87^ and Rung-3.5 functionals^89,90^ use the density coherence as an ingredient). Note that in the coordinate representation, the density coherence between electrons at spatial coordinate **r** and **r′** can be written as *ρ*(**r**|**r′**) with **r** ≠ **r′**, and the density at **r** is

8.1 *ρ*(**r**) = *ρ*(**r**|**r**)

Because a multiconfigurational wave function improves the density coherence,^85^ the density coherence provides an alternative route to developing accurate nonclassical-energy functionals. Motivated by the above considerations, we recently developed a new method named multiconfiguration density coherence functional theory (MC-DCFT), which is a special case of MC-NEFT.^26^

In MC-DCFT, a density-coherence functional *E*_dc_[***ρ***] is used as the nonclassical-energy functional. The MC-DCFT energy is therefore defined as

8.2 *E*_MC-DCFT_ = *E*_MC,class_ + *E*_dc_[*ρ*(**r**|**r′**)]

where *E*_MC,class_ is the classical energy of the multiconfiguration wave function.

In the original version of MC-DCFT,^26^ we construct the density coherence functional based on the unpaired density,^65,91,92^ which is defined as8.3
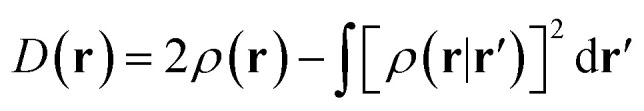


Like “translated” functionals in MC-PDFT,^7^ we then define the effective spin density of the major- and minor-spin electrons as8.4
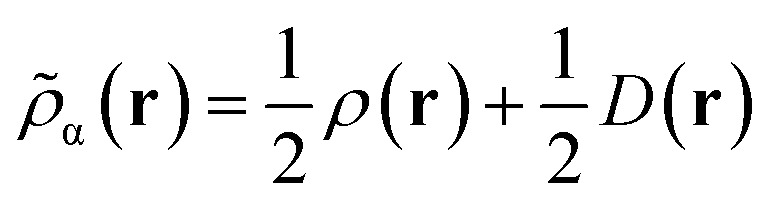
8.5
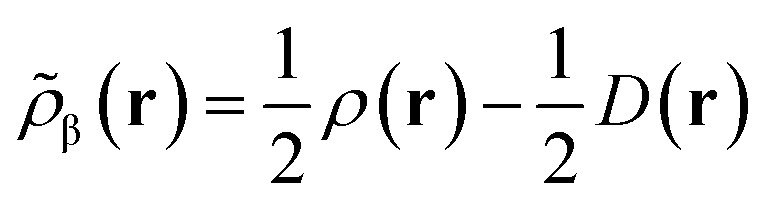


The effective spin densities obtained this way differ from the ones obtained by translating the density and on-top pair density, but are used in the same way, *i.e.*, by employing them as the effective spin densities in an existing KS functional to evaluate the total MC-DCFT exchange and correlation energy. Density coherence functionals developed this way are called converted (“c”) functionals. *e.g.*, cPBE.

**Fig. 5:**
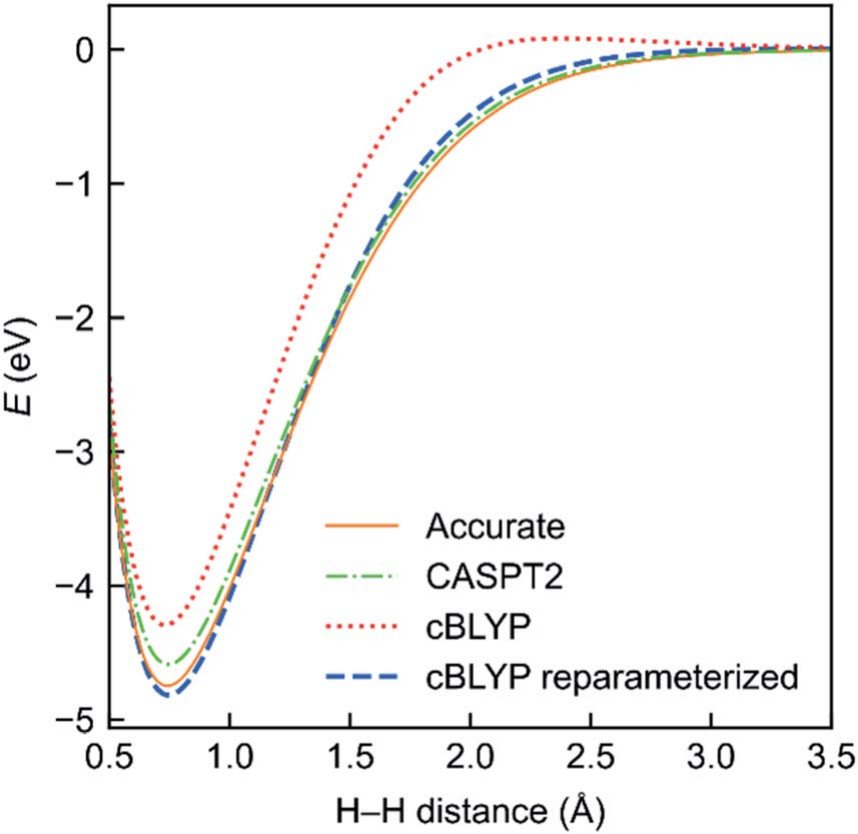
Potential energy functions of H_2_ using the cBLYP density coherence functional with and without reparameterization − as compared to the accurate and CASPT2 potential energy functions.

To evaluate the performance of the MC-DCFT method, we calculated the potential energy curves of H_2_, F_2_, N_2_, and HF molecules;^26^ the results for H_2_ are shown in Fig. 5. We found that all potential energy curves of the original converted functionals that we tested contain systematic errors; however, a two-parameter reparameterization reduced the errors significantly. This shows that MC-DCFT has promise for a wide range of applications if one fully optimizes the density coherence functional. We are currently carrying out such optimization.

## 9. Machine-learned functionals

Currently the only functionals commonly in use for MC-PDFT are those that have been translated from KS-DFT, mainly translated PBE (tPBE). While both HMC-PDFT and MC-DCFT have been exciting research directions for MC-NEFT functional development, they do not present a fundamental departure from the translation approach as their energies are ultimately calculated *via* the input of derived features into standard KS functional forms. Despite this, as far back as the initial publication of MC-PDFT in 2014 (ref. 7) we have stated that “ultimately, we must develop new on-top functionals specifically for use with MC-PDFT”.

Our recent investigations into machine-learned functionals for MC-NEFT present what is finally a true departure from the functionals of KS-DFT, allowing a direct utilization of, in principle, all information contained within the multiconfigurational wave function. While this research has paralleled the exciting development of machine-learned functionals for KS-DFT,^93–97^ machine-learning approaches are particularly well-suited for MC-PDFT because (1) unlike KS theory, MC-PDFT does not benefit directly from decades of research into physically motivated functional forms; and (2) MC-PDFT predicts energies based on features of qualitatively correct multiconfigurational wave functions, which one would anticipate to greatly simplify the learning problem as compared to leaning from a sometimes unphysical Kohn–Sham determinant.^93^ Furthermore, the multiconfigurational wave function in MC-PDFT contains vastly more information than the density in KS-DFT.

Recall that in Section 3 we defined the PDFT energy as the sum of a classical energy and a nonclassical energy. Broadly speaking, in the machine-learning approach one tries to approximate the nonclassical energy as a parameterized function *E*^nc^_ML_ of some featurization *f* of the multiconfigurational wave function,

9.1 *E* = *E*_class_[*ψ*^MC^] + *E*^nc^_ML_[*f*[*ψ*^MC^]]

In principle, this featurization is very general, and it can involve any information available in *ψ*^MC^: the density, on-top density, density coherence, gradients of these quantities, and/or potentially even information from the 2- or 3-body RDMs. In our first approach, we have featurized the wave function atom-wise (following the KS-theory work of Dick and Fernandez-Serra^93^) by projecting *ρ*^MC^ and Π^MC^ onto atom-centered featurization basis functions *ϕ*^*I*^_*a*_ where *I* specifies the atom:9.2
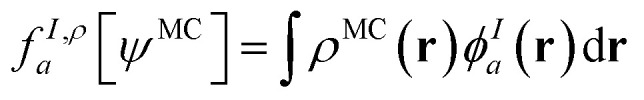
and9.3
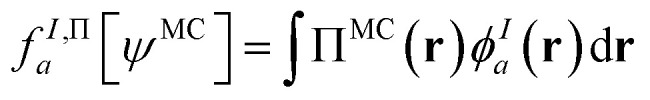


These projections were then made rotationally invariant, resulting in a total of 72 features per atom, {*g*^*I*,ρ^_*a*_,*g*^*I*,Π^_*a*_}. These atomic features served as input into a Behler–Parrinello-type neural network,^93,98^ outputting a size-extensive prediction of the nonclassical energy:9.4
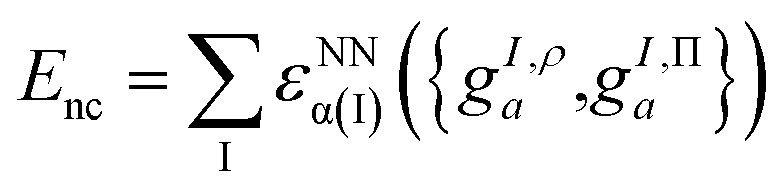
where *ε*^NN^_α(I)_ is an element-dependent fully connected neural network. This atom-based featurization parallels the use of grid-based featurization in conventional KS theory, although one benefit of the atom-based approach is that in principle it avoids the scaling of computational costs with respect to grid size (and is instead dependent on the number of atomic input features).

Our initial generation of machine-learned nonclassical-energy functional theory (ML-NEFT) was trained on the problem of predicting carbene singlet–triplet energy gaps using the recently published *QMSpin* database,^99^ and we obtained mean absolute errors less than 0.05 eV on test data with a robust degree of active space independence.^100^ While these results provide a proof of concept, obtaining a broadly useful new functional will require the training and test data to be expanded to a much larger range of data, and we are currently pursuing this direction.

Machine-learned functionals can also be used with other ingredients or used in other combinations with multiconfigurational wave functions to obtain even more general energy functionals for strongly correlated systems.

## 10. Multistate pair-density functional theory

For a balanced treatment of ground and excited states, one can use a state-averaged (SA) wave function as a reference for MC-PDFT. This approach was found to be as accurate as CASPT2 for a set of 23 vertical excitation energies^101^ and consistent with CASPT2 in reproducing the trends in vertical excitation energies of rhodopsins.^102^ It has also been used successfully for other problems, as reviewed elsewhere.^10^ However, sometimes it is not sufficient to calculate the final state energies independently, even if one uses state-averaged orbitals. A major motivation in developing MC-PDFT (or any electronic structure method) is to model molecular dynamics. Photochemical dynamics is often dominated by regions of coordinate space where potential energy surfaces approach closely,^103,104^ a situation that is sometimes called quasidegeneracy. A practical treatment of quasidegenerate states requires state interaction.^105^ State interaction requires the final approximations to the electronic states of interest to be simultaneous eigenvectors of the same Hamiltonian matrix. State interaction is required for two reasons: (i) it gives the final potential energy surfaces the correct topology at the conical intersections; (ii) it produces a set of mutually orthogonal electronic states that can be used for multistate dynamics calculations.

Single-state MC-PDFT, which is what we have been discussing so far, does not provide state interaction because, even if the orbitals are optimized by SA-CASSCF, the last steps of the MC-PDFT calculations on the various states are independent rather than corresponding to the diagonalization of an effective Hamiltonian matrix. For multistate wave function methods such as extended multiconfiguration quasidegenerate perturbation theory (XMC-QDPT),^106^ extended multistate CASPT2 (XMS-CASPT2),^107,108^ or multireference *N*-electron valence state perturbation theory with quasi-degenerate perturbation theory (NEVPT2/QDPT),^109^ the final step is the diagonalization of an effective Hamiltonian called the model-space Hamiltonian, which is an *n* × *n* matrix, where *n* is the number of interacting adiabatic states of interest, which may be as small as two. Constructing an analogous model-space Hamiltonian with MC-PDFT requires a new kind of blending of the wave function and density functional methods because the energy expression used in MC-PDFT is defined only for use in diagonal matrix elements, not for use in matrix elements connecting different CSFs or different states in the model space. To produce model-space Hamiltonians with MC-PDFT, we proposed multistate pair-density functional theory (MS-PDFT).^61,110^ (This was included very briefly in our previous review;^9^ here we give a more complete discussion). In MS-PDFT, the final step is the diagonalization of an *n* × *n* model-space Hamiltonian.

A key step in multistate methods is the choice of state basis for the model space. This step is very similar to the first step in conventional degenerate perturbation theory,^111^ and in fact, perturbation theories employing a model space are often called quasidegenerate perturbation theory.^105,109,112^ In MS-PDFT, the basis used for the model space is called the intermediate basis; it is a basis consisting of *n* linear combinations of *n* SA-CASSCF eigenstates, but a choice must be made to decide on which linear combination to use. This choice is particularly crucial in MS-PDFT because the PDFT energy functional will be applied to the model-space basis functions to produce the diagonal elements of the model-space Hamiltonian. The goal is to find an intermediate basis that makes the resulting diagonalization as accurate as possible. We found that a good choice of an intermediate basis is the one that maximizes the sum over the intermediate states of the classical Coulomb energy10.1
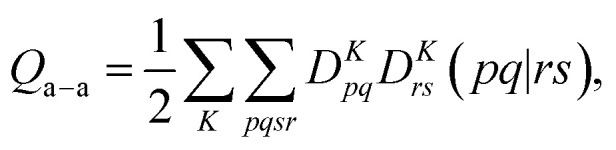
where *D*^*K*^_*pq*_ is the one-electron density matrix for an intermediate state *K*. Maximizing this term is equivalent to maximizing the sum over states of the classical electron–electron Coulomb repulsion energies, and the intermediate states obtained by doing so have more compact electronic densities than the starting SA-CASSCF states. This method is called compressed multistate PDFT (CMS-PDFT). The physical motivation is that approximate density functionals are more accurate on compressed electron densities, and the possibility of overcounting correlation energy in the model-space diagonalization is reduced with this choice. The compressed intermediate states are obtained by transforming the SA-CASSCF wave functions, which are also called reference wave functions, to the intermediate basis:10.2
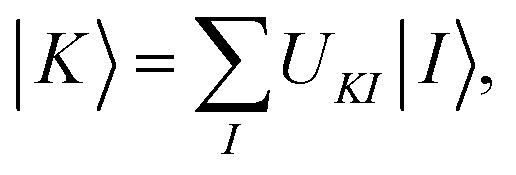
where |*K*〉 is an intermediate state, |*I*〉 is an SA-CASSCF eigenstate, and *U*_*KI*_ is an element in the rotation matrix **U**. With the intermediate states, we build an effective Hamiltonian matrix, whose diagonal elements are the MC-PDFT energies for the intermediate states, and the off-diagonal elements are the couplings computed by the wave function method,10.3
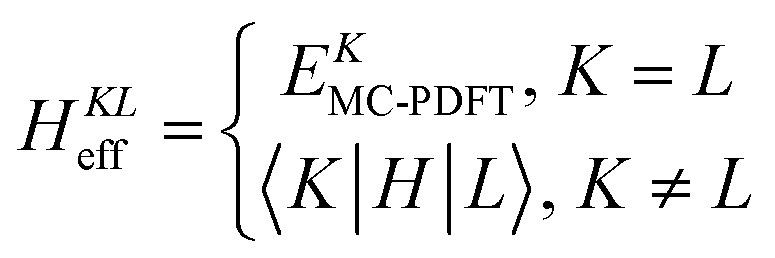


The CMS-PDFT final states and energies are obtained by diagonalizing the effective Hamiltonian matrix.

In earlier work, we also considered other ways to obtain **U**, each leading to different versions of MS-PDFT, as discussed next. In each of these, the final states and energies are obtained by diagonalization of an effective Hamiltonian matrix.

Extended multistate PDFT (XMS-PDFT) uses the same intermediate states as are used in XMC-QDPT and XMS-CASPT2. The motivations for using these intermediate states are^106^ (1) to eliminate overestimation of the off-diagonal elements of the effective Hamiltonian and (2) to define intermediates states that are invariant to arbitrary transformations of the SA-CASSCF states.

In variational multistate PDFT (VMS-PDFT), the intermediate states are chosen to maximize the trace of the effective Hamiltonian matrix, namely the sum of the MC-PDFT energies for the intermediate states,10.4
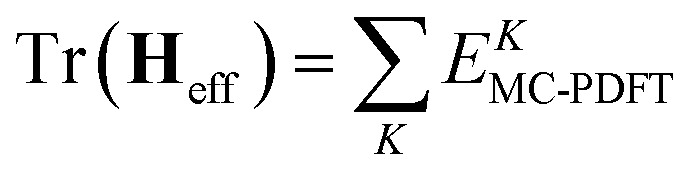


The physical motivation in VMS-PDFT is that, by maximizing the sum of MC-PDFT energies, the contribution of near-degeneracy correlation energy to the PDFT energy functional is removed to the maximum degree possible. The remaining near-degeneracy correlation energy is then recovered by diagonalizing the effective Hamiltonian matrix. One way to maximize the trace is to expand the transformation matrix **U** of VMS-PDFT with a Fourier series; this is called Fourier-series multistate PDFT (FMS-PDFT). The motivation is to reduce the cost of maximizing the trace of the effective Hamiltonian matrix.

We have tested all four methods on a series of systems. We found that VMS-PDFT, FMS-PDFT, and CMS-PDFT work for all systems tested. However, XMS-PDFT (which is the least expensive alternative), although usually successful, failed for a mixed-valence system, the 2,2′,6,6′-tetrahydro-4*H*,4′*H*-5,5′-spirobi[cyclopenta[*c*]pyrrole] cation. Among VMS-PDFT, FMS-PDFT, and CMS-PDFT which are all robust, CMS-PDFT is the most efficient. Because the CMS-PDFT method has the best combination of computational efficiency and robust performance, it is the method we have selected for further development for photochemical applications.

Before proposing the MS-PDFT methods, we proposed a model-space method called state-interaction PDFT^113^ (SI-PDFT). Although this method was successful, it is computationally less convenient than MS-PDFT. It is compared to MS-PDFT in Appendix B.

Another similar method is multistate density functional theory^114–120^ (MSDFT). This method is like MC-PDFT in that both kinds of method have a state-interaction step “after”^121^ the dynamic correlation is included. In MSDFT, this uses the overlap of two states to compute the off-diagonal elements of the Hamiltonian.^118^ However, this equation does not apply to MS-PDFT because the MS-PDFT intermediate states are mutually orthogonal.

## 11. Spin–orbit coupling

Spin–orbit coupling (SOC) is of great importance for understanding intersystem crossing,^122^ phosphorescence,^123^ magnetic anisotropy,^124,125^ magnetic qubits for quantum computers,^126,127^ and chemical reactions.^128–131^ One of the main difficulties in computing SOC effects is to combine methods for accurate energies of the spin–orbit free states with the consistent treatment of SOC. A conventional procedure for transition-metal compounds or molecules containing lanthanide elements is to perform such calculations by multireference perturbation theory. However, just as we have seen in previous sections of this perspective, our goal is to obtain comparable or better accuracy in a more affordable way that applies to large and complex molecular systems.

MC-PDFT provides an efficient routine to obtain accurate excitation energies, and MS-PDFT methods can include the necessary state interaction at a low computational cost. The SOC-inclusive Hamiltonian is the sum of a diagonal spin-free Hamiltonian composed of MC-PDFT or MS-PDFT energies and a nondiagonal Hamiltonian containing the matrix elements of the spin–orbit operator. A key issue is how the SOC-inclusive Hamiltonian is formed and diagonalized. For the SOC calculations with MC-PDFT, the basis for the SOC-inclusive Hamiltonian is the SA-CASSCF eigenvectors. For SOC calculation with MS-PDFT methods (such as XMS-PDFT and CMS-PDFT), the basis sets are the states that diagonalize the effective Hamiltonian mentioned in the previous section.

For PDFT calculations of the Zeeman effect of the ground state, we also proposed a complete-active-space configuration interaction (CASCI) scheme, which is a systematic way to improve the results of the spin–orbit coupling calculations by using orbitals optimized just for the ground state.^132^ For the calculation of zero-field splitting of the ground state, a particularly successful strategy is to use weighted state-averaged CASSCF (WSA-CASSCF) orbitals in which the ground state has an especially high weight (rather than the conventional choice of equal weights for all states averaged).^133^

We combined previously developed methodology^134^ with SOC-inclusive PDFT calculations to calculate both the Zeeman effect (as described by the ***g*** tensor^135,136^) and the zero-field splitting (described mainly by the zero-field splitting parameter *D*^135,137^) of transition-metal complexes. The accuracy of utilizing this combined method has been validated by comparing ***g*** tensors^132^ and *D* parameters^133^ to experiment. We showed that MC-PDFT and XMS-PDFT agree with CASPT2 for Zeeman effect calculation, and CMS-PDFT gives useful accuracy for zero-field splitting parameters, although the cost of PDFT based methods is much smaller than CASPT2 in both cases. MC-PDFT has also been used to calculate the SOC effect in Ce^+^ and CeH^+^, and MC-PDFT and XMS-PDFT provide a spin–orbit energy closer to the experimental value than does SA-CASSCF.^138^

In two recent articles,^139,140^ we have used MC-PDFT to study the photodynamics of SOC-promoted intersystem crossing. A greater range of photochemical processes will soon be available to PDFT methods because our group has recently completed the coding for efficient CMS-PDFT analytic gradients, as discussed in the next section.

## 12. Forces by analytic gradients

Forces on the nuclei are given by the negative gradients of the potential energy surfaces. These gradients are also used for geometry optimization. Analytic gradients are much more efficient than numerical gradients, and the development of analytic gradient methods for variational energies^141^ is one of the major advances that made electronic structure calculations a powerful tool in many branches of chemistry. For nonvariational energies, such as those predicted by MC-PDFT and MS-PDFT, one may obtain gradients by a Lagrangian method,^142–145^ and that is used here.

We presented analytic gradients for MC-PDFT in a series of three papers concerned successively with MC-PDFT based on CASSCF orbitals,^146^ MC-PDFT based on SA-CASSCF orbitals,^147^ and MC-PDFT with the calculation of two-electron integrals speeded up by density fitting using Cholesky decomposition.^148^

The Lagrangian for the *I*th root of an MC-PDFT calculation based on SA-CASSCF orbitals is12.1
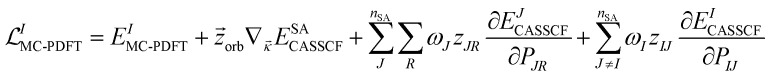
where *E*^*I*^_MC-PDFT_ is the MC-PDFT energy for state *I*, *E*^*J*^_CASSCF_ is the SA-CASSCF energy for state *J*, *n*_SA_ is number of states in the average, *P*_*JR*_ is the configuration interaction (CI) transfer parameters of state *J*, an active-space state that appears in the state average, to state *R*, an active space state that does not appear in the state average, *ω*_*I*_ is the weight of state *I*, and *κ⃗* denotes the orbital rotation parameters for general orbitals. The *z* terms with subscripts *JR*, *IJ*, and orb are Lagrange multipliers for CI transfers outside the state-averaged space, CI transfers within the state-averaged space, and orbital rotation parameters, respectively. With a set of Lagrange multipliers that satisfy appropriate conditions, one can differentiate eqn (12.1) with respect to an external perturbation, *λ*.

In *PySCF* and *OpenMolcas* the analytic gradient of the energy with respect to nuclear coordinates has been implemented with density fitting to avoid the bottleneck associated with the construction of the two-electron integrals and their transformation into the atomic orbital basis. MC-PDFT with density fitting is critical to optimizing the geometry of larger systems with both state-specific and state-average MC-PDFT. For example, we considered rhodopsin (49 atoms) with a (12,12) SA-CASSCF calculation and the cc-pVDZ basis set. Without density fitting we were not able to optimize the geometry of this system; with density fitting, the optimized geometry is similar to the CASPT2 optimized geometry.

The analytic gradients for SA-PDFT have been used recently in dynamics simulations of the thioformaldehyde system. The gradients of the two lowest-lying states in the singlet and triplet manifolds were used to determine the populations of each of the states for the dynamic simulations. The MC-PDFT dynamics simulations are an improvement on the SA-CASSCF dynamics simulations, which showed unphysical behavior, and are in good agreement with CASPT2 dynamics.

The above development of the state-specific analytic gradients was a first step in obtaining the dipole moments discussed in the next section.

Next, we developed analytic gradients for the CMS-PDFT version of MS-PDFT, and we implemented them in both *PySCF* and *OpenMolcas.*^149^ In CMS-PDFT analytic gradients, there are energy parameters for the final CMS eigenvectors, the intermediate state transfers, the orbital rotation operators, and the CI transfers outside of the state-averaged manifold. Since the last step in CMS-PDFT diagonalizes the effective Hamiltonian matrix, we do not need Lagrange multipliers for the final diagonalization. The general form of the Lagrangian for the CMS-PDFT is12.2
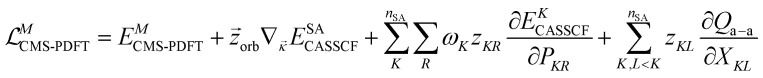
where *K* and *L* are the CMS intermediate states, *M* is the CMS-PDFT final state, *E*^*M*^_CMS-PDFT_ is the energy of the final CMS-PDFT state, *X*_*KL*_ is for state transfers from an intermediate state to an intermediate state, and *Q*_a–a_ was introduced in Section 10. Then one can solve for all the Lagrange multipliers, *z*, and take the derivative of the Lagrangian.

The gradients for the CMS-PDFT method are useful for applications with nearly degenerate electronic excited states, such as systems that are near conical intersections or locally avoided crossings. For instance, the equilibrium geometry of the first singlet excited state of the phenol molecule lies near an S_1_–S_2_ conical intersection.^150^ Test calculations indicate that CMS-PDFT is a promising method for studying excited states with strong state interaction.^149^

Having efficient gradients for CMS-PDFT makes possible the simulation of a wide variety of photochemical and other electronically nonadiabatic processes using curvature-driven semiclassical methods.^151^

## 13. Dipole moments

The electric dipole moment is the leading nonzero moment of the charge distribution in a neutral molecule. It is especially important for controlling the infrared activity of vibrations^152,153^ molecular recognition,^154^ and the strength of long-range noncovalent interactions.^155,156^ In general, the permanent dipole moment can be formulated either as the expectation value of the dipole moment operator or as a response, where the response is the energy derivative with respect to the electric field strength.^157^ These two formulations are identical for the exact wave function, but the response formulation is more accurate for approximate wave functions. Furthermore, the response formulation is the only one available for MC-PDFT because MC-PDFT evaluates the energy without producing a wave function corresponding to that energy.

To evaluate MC-PDFT dipole moments, we performed analytic differentiation of the MC-PDFT energy using Lagrange's method of undetermined multipliers.^158^ This approach shares the same set of Lagrange multipliers as discussed in the previous section. Since the HMC-PDFT energy is a linear combination of the CASSCF and MC-PDFT energies, the HMC-PDFT analytic dipole moment comes with no additional cost compared to MC-PDFT. In fact, we showed that the HMC-PDFT analytic dipole moment is simply a sum of the CASSCF dipole and the PDFT correction expressed in terms of the Lagrange multipliers13.1
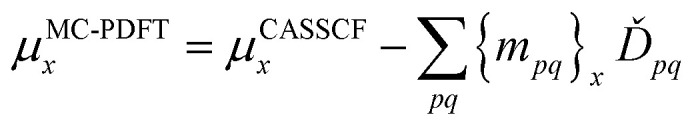
13.2
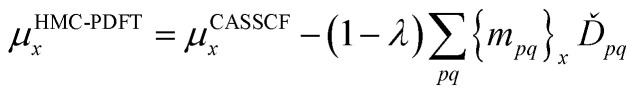
where *μ*_*x*_ is the component of the dipole moment along the *x*-axis, *m*_*pq*_ is an electric dipole moment integral, and *Ď*_*pq*_ is an element of an effective one-electron reduced density matrix that depends on the Lagrange multipliers.

To assess the performance of MC-PDFT for predicting dipole moments, we first explored dipole moments of diatomic molecules as functions of geometry.^158^ Using a CASSCF calculation with a full-valence active space for the first step, we compared MC-PDFT with the tPBE on-top functional and HMC-PDFT with the tPBE0 functional to MRCI with single and double excitations and a quadruples correction (MRCISD + Q). For HF, CO, and NO, we found that the MC-PDFT and HMC-PDFT dipole moments were much closer to MRCI + Q than to CASSCF. These are especially interesting tests because HF is about 50 : 50 ionic and covalent and CO and NO both change their polarity as functions of internuclear distance. A similar check for AlO found that four considered multireference methods gave similar results for both the ground and first excited state.

**Fig. 6:**
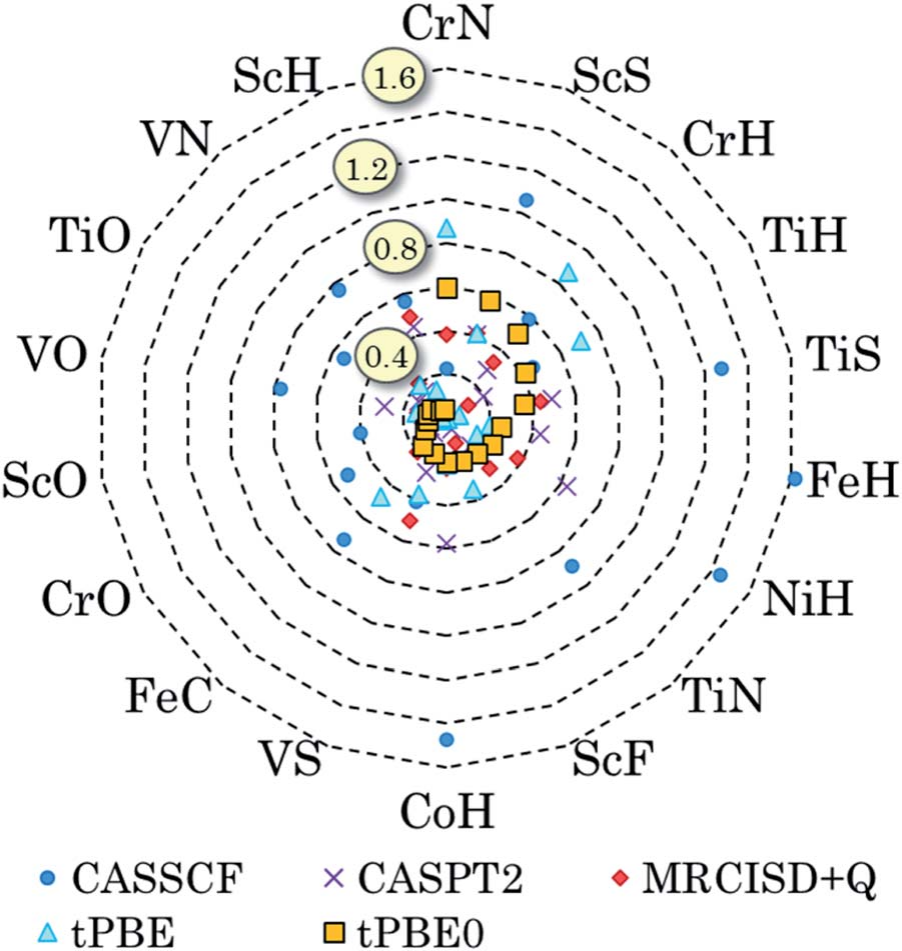
Unsigned errors in equilibrium dipole moments (in debye). All diatomics have a multireference character, except ScF. All methods share the same CASSCF wave function for a given molecule. Adapted with permission from ref. 158.

We also tested MC-PDFT and HMC-PDFT for 18 diatomics containing a transition metal and H, C, N, O, or S.^158^ We used the moderate correlated participating orbitals^43^ (*mod*-CPO) active space prescription. The mean unsigned error of the various methods is as follows: 0.55 D for CASSCF, 0.29 D for MC-PDFT with tPBE, 0.24 D for HMC-PDFT with tPBE0, and 0.28 and 0.25 D respectively for the much more expensive CASPT2 and MRCISD + Q calculations. These errors for individual molecules are shown in Fig. 6. These are particularly interesting tests because 17 of the 18 molecules (all except ScF) are strongly correlated, as judged by the *M* diagnostic^43^ of multireference character.

## 14. Concluding remarks

Kohn–Sham density functional theory with available functionals is very powerful but is more accurate for weakly correlated molecules and transition states than for strongly correlated ones. Most excited states, systems with stretched bonds, and most catalytic intermediates are strongly correlated. Proper treatment of strongly correlated systems requires incorporating multiconfigurational character into the representation of the density. Multiconfiguration nonclassical-energy functional theory (MC-NEFT), of which multiconfiguration pair-density functional theory (MC-PDFT) is the most developed special case, combines multiconfigurational wave functions with a generalization of density functional theory that is especially designed to treat molecules with strong correlation. For multireference systems, multi-configuration self-consistent field (MCSCF) calculations, *e.g.*, complete-active space self-consistent field (CASSCF) calculations, can produce a density that is much more accurate than the density produced by the Hartree–Fock single configuration method, but they are usually quantitatively inaccurate for energetics due to incomplete treatment of the correlation energy, which is very slowly convergent with respect to the number of configuration state functions included. For quantitative work with predictive accuracy for energetics, one must therefore follow the MCSCF calculation with a post-SCF energetic calculation such as multireference perturbation theory (MRPT) or multireference configuration interaction (MRCI), but these traditional approaches are more expensive than adding a density functional step, and they are often impractical for complex systems.

In MC-NEFT, an MCSCF wave function with the correct spin symmetry – and optionally also the correct spatial symmetry – is calculated first, but the post-SCF steps of MRPT or MRCI are replaced by an inexpensive calculation involving a functional of the kinetic energy, density, and other features in the MCSCF wave function. These other features are the on-top pair density and optionally its gradient in MC-PDFT, the on-top pair density, the MCSCF energy, and optionally the gradient of the on-top pair density in hybrid MC-PDFT (HMC-PDFT), the density coherence in multiconfiguration density-coherence functional theory (MC-DCFT), and any convenient features of the MCSCF wave function when a nonclassical-energy functional is parameterized by a neural network.

To treat closely coupled states, such as interacting nearly degenerate states, we also developed a multistate generalization by compressed multistate (CMS) formalism that includes state interaction, as required to treat molecules near conical intersections. These methods are all more affordable than wave function theories like MRPT and MRCI, and they are already allowing applications to difficult systems that are hard to treat in any other affordable way.

Two major difficulties of MC-PDFT are the choice of active space and the quality of the density functionals. The active space choice is a problem shared by all methods that use multiconfiguration reference functions, namely, how to ensure that the active space is balanced, how to systematize the choice of active space, and how to afford the cost and required computer resources of large active spaces when they are necessary. We have made progress in all three areas, but further work will be valuable. Generalizing MC-PDFT to nonclassical energy functional theory is a direction we have started for improving the density functionals; another direction will be including additional ingredients like kinetic energy density.

## Appendix A

This appendix gives some of the equations of MC-PDFT to provide a more mathematical background for material discussed in broader terms in the main text of this perspective.

Multiconfiguration nonclassical-energy functional theory (MC-NEFT) denotes the set of electronic structure methods in which the classical components of the electronic energy (kinetic energy, electron–nuclear attraction, and classical electron–electron interactions) are treated with wave function theory, using a multiconfiguration reference wave function *ψ*^MC^, while the nonclassical components (exchange and correlation) are treated with a functional *f* of the multiconfiguration reference wave function. In its most general form, the MC-NEFT energy is written as

A-1 *E*_MC-NEFT_[*ψ*^MC^] = *E*_class_[*ψ*^MC^] + *E*_nc_[*f*[*ψ*^MC^]],

where *E*_class_ is the classical energy, and *E*_nc_ is the nonclassical energy depending on a functional, which is called the on-top functional in MC-PDFT [see eqn (3.1)], the density-coherence functional in MC-DCFT [see eqn (7.2)], and the machine-learned functional in ML-NEFT [see eqn (9.1)]. The MC-PDFT method is the original and still most widely studied of the MC-NEFT methods; in MC-PDFT, the on-top functional for state *I* (*i.e.*, for *ψ*^MC^ = *ψ*_*I*_) is computed as a functional of the density *ρ*_*I*_ and the on-top pair density Π_*I*_. The on-top pair density is defined byA-2
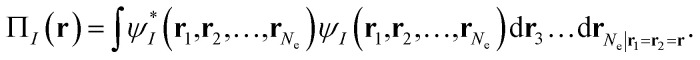


Note that the density is defined as the probability density to find an electron at a point **r** in space, and the on-top pair density is defined as the probability density to find two electrons at a point **r** in space.

In work so far, the on-top functional *E*_OT_[*ρ*(**r**),Π(**r**)] is usually defined by “translation” or “full translation” of a Kohn–Sham exchange–correlation functional *E*_XC_(*ρ*,*m*,*ρ*′,*m*′) that depends on the density *ρ*(**r**), the spin magnetization density *m*(**r**), and the magnitudes of their gradients, *ρ*′(**r**) and *m*′(**r**). Equations are given in the original articles, but here we give, as an example, the equation for simple translation:A-3
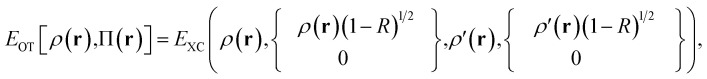
where the upper level of the brace is used for *R* ≤ 1 and the lower level for *R* > 1, where

A-4 *R* = Π(**r**)/[*ρ*(**r**)/2]^2^

It is worth emphasizing the difference in the way that reference functions are used in the various theories considered in this article. In multireference wave function theory and in MC-PDFT, the reference wave function is multiconfigurational and is an approximate wave function of the real system. In KS theory, the reference wave function is single-configurational (a Slater determinant) and is the real wave function of a model system defined in terms of the real system (the model system is a system of noninteracting electrons having the same density as the real system). When we recall, though, that we do not have exact density functionals for KS theory, we see that in practical calculations the Slater determinant is the real wave function of a model system defined in terms of an approximation to the real system. Despite this difference between KS theory and MC-PDFT, in both cases we compute the one-electron energy terms (kinetic energy and nuclear attraction) from the reference function.

## Appendix B

This appendix compares the adopted MS-PDFT methods of Section 10 to the earlier state-interaction PDFT method (SI-PDFT).

The on-top energy of each state in MC-PDFT is calculated separately according to the density and on-top density of each state, and therefore MC-PDFT includes no interaction between electronic states and fails to give the correct topography of potential energy surfaces at conical intersections. This failure is remedied by multi-state PDFT (MS-PDFT), which is discussed in Section 10, and also by the earlier SI-PDFT. In SI-PDFT, the reference SA-CASSCF excited states {*ψ*^SA^_*I*_} and an auxiliary state *ψ*^SS^ from a state-specific CASSCF (SS-CASSCF) ground-state calculation are generated to construct an intermediate-state basis {*Θ*_*I*_}. The ground intermediate state is obtained by projecting the SS-CASSCF ground state into the space spanned by the SA-CASSCF states asB-1
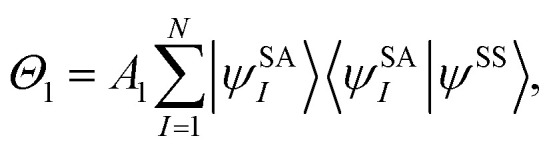
where *A*_1_ is the normalization coefficient for *Θ*_1_. The other (*J*–1) intermediate states are constructed to be orthogonal to each other byB-2
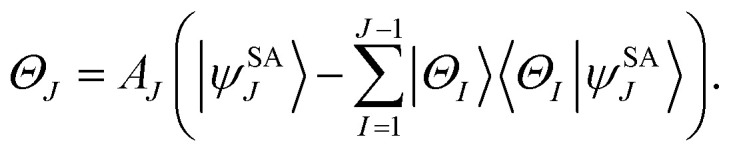


The above construction of intermediate states in SI-PDFT plays the same role as the rotation in eqn (10.2); however, in SI-PDFT, the intermediate states are obtained with two sets of orbitals, *i.e.*, one orbital set for the SS-CASSCF ground state and another orbital set for the SA-CASSCF excited states. Therefore, a biorthogonal transformation is used to construct **U**, and this causes greater complexity and higher computational costs. A second difference is that SI-PDFT treats the ground state differently from the excited states, and this could be troublesome if there is a conical intersection of the ground state with an excited state, whereas MS-PDFT treats all states on an equal footing. For these two reasons, we prefer the later multistate methods, denoted VMS-PDFT, FMS-PDFT, XMS-PDFT, and CMS-PDFT.

## Appendix C

This appendix discusses the treatment of the kinetic energy in MC-PDFT and compares this treatment to KS theory.

It has been suggested that it would be instructive to analyze the components of the MC-PDFT electronic energy by considering how it approximates the kinetic energy. Since the kinetic energy term used in MC-PDFT is not the kinetic energy of the exact wave function and because we do not have an exact on-top functional for a given choice of multiconfiguration wave function, one might say that the available on-top functionals do not calculate the correction to the inexact kinetic energy perfectly, but this is hard to quantify because one does not separate the on-top energy into kinetic and potential energy components. A similar problem occurs in KS theory where the explicit kinetic energy term is not the kinetic energy of the exact wave function. Because we do not have an exact exchange–correlation functional in KS theory for a general system, one may say that the available exchange–correlation functionals do not calculate the correction to the inexact kinetic energy perfectly, but this is hard to quantify because one does not separate the exchange–correlation energy of practical calculations into kinetic and potential energy components. This separation is straightforward in a variational wave function calculation because the electronic energy in such a case is the expectation value of the sum of a kinetic energy term and a potential energy term, but it is not straightforward in a calculation employing an approximate density functional.

Even though one does not usually explicitly separate the kinetic and potential energies in practical density functional calculations, one can still make some comments about the implicit kinetic energy. One can first notice that although KS theory uses the kinetic energy of the optimized single-configuration wave function, that kinetic energy would not be the expectation value of the kinetic energy operator for the exact wave function even if one were able to use the (unknown) exact functional. The exchange–correlation functional must therefore make up for the difference in the kinetic energy of the Slater determinant and the kinetic energy of the (unknown) exact wave function as well as making up for the difference in potential energy due to exchange and correlation. The difference of the kinetic energy of the Slater determinant from the (unknown) exact kinetic energy is sometimes called a correlation effect, but it is different from the correlation effect on the kinetic energy in wave function theory; in wave function theory the correlation kinetic energy would be the difference between the Hartree–Fock kinetic energy and the kinetic energy operator for the exact wave function.

Similarly, in MC-PDFT, the kinetic energy of the multiconfiguration wave function is not the expectation value of the kinetic energy operator for the exact wave function. In addition, the classical electrostatic energy of the multiconfiguration wave function is not the same as the classical electrostatic energy computed from the exact density. The on-top functional must make up for these differences as well as for exchange and correlation contributions to the energy, and in fact one long-term goal would be to design a functional that does this for a given systematic prescription for which configurations to include in the multiconfiguration wave function. However, even in the absence of functional that accomplishes this design objective, the kinetic energy difficulty should be reduced as compared to KS theory because the kinetic energy of the multiconfiguration wave function should be closer to the exact kinetic energy than is the kinetic energy of the single-configuration reference wave function of KS theory. Nevertheless, the main reason that the energy from MC-PDFT should be more accurate than the energy from KS theory, with equally well-developed functionals, is that the classical Coulomb energy and the electron density should be more accurate because MC-PDFT uses information about a correlated calculation of the density and on-top pair density.

## Data availability

Since this is a review-and-perspective article, we refer readers to the original publications for data.

## Author contributions

All authors contributed to the research that is reviewed here. All authors wrote part of the first draft, and all authors contributed to editing the manuscript. LG and DGT were responsible for funding acquisition, project management, and supervision.

## Conflicts of interest

There are no conflicts to declare.

## Supplementary Material
